# tRNA-derived fragment *tRF-24* drives CELF1 phase separation to promote oncogenic splicing in esophageal squamous cell carcinoma

**DOI:** 10.1186/s13046-025-03553-x

**Published:** 2025-11-17

**Authors:** Yajie Hu, Xin Qin, Li Gong, Ling Pan, Yufeng Cheng

**Affiliations:** 1 Department of Radiation Oncology, Qilu Hospital of Shandong University, Cheeloo College of Medicine, Shandong University, Jinan, China; 2 Shandong Provincial Key Laboratory of Malignant Tumor Precision Treatment, Jinan, China; 3 Shandong Provincial Engineering Research Center for Tumor Precision Treatment, Jinan, China; 4 Yellow River Basin Collaborative Innovation Center for Precision Oncology, Jinan, China; 5 Cancer Institute of Shandong University, Jinan, China; 6 Neutron Medical Center, Qilu Hospital of Shandong University, Jinan, China; 7 Departmen of Urology, Qilu Hospital of Shandong University, Cheeloo College of Medicine, Shandong University, Jinan, China

**Keywords:** TRF-24, CELF1, Phase separation, Alternative splicing, Post-translational modification, ESCC

## Abstract

**Rationale:**

Esophageal squamous cell carcinoma (ESCC) is characterized by poor prognosis. tRNA-derived fragments (tRFs), a novel class of non-coding RNAs generated by tRNA cleavage, have emerged as key regulators of cancer development. However, the functional landscape of tRFs remains underexplored in ESCC. We here identified *tRF-24-RPM8309M2S* (*tRF-24*), a 5’ tRF derived from mature *tRNA*^*LeuAAG/TAG*^, which promotes the malignant progression of ESCC and offers a promising therapeutic target.

**Methods:**

The public GSE207635 dataset from Gene Expression Omnibus (GEO) database was analyzed to identify tsRNAs involved in ESCC progression. The clinical significance of *tRF-24* was investigated in samples from 96 ESCC patients. CUGBP Elav-like family member 1 (CELF1) was validated as a *tRF-24* interactor through RNA pull-down assays. CCK-8 and transwell assays were applied to measure malignant cell phenotypes. mCherry-GFP-LC3 reporter assay was performed to examine the autophagy. Colocalization between LC3 and mitochondria was employed to detect mitophagy. Immunofluorescent and colony formation assay were conducted to assess the impact of DNA damage repair and cisplatin resistance in ESCC. Extracellular acidification rate (ECAR), lactate production and glucose consumption were performed to analyze changes in glycolysis. Fluorescence recovery after photobleaching (FRAP) was adopted to evaluate CELF1 phase separation. Additionally, RNA sequencing and alternative splicing analyses were conducted to determine global transcriptome alterations following *tRF-24* or CELF1 overexpression.

**Results:**

Our findings demonstrate that *tRF-24* is significantly upregulated in tumor samples and is associated with poorer survival of ESCC patients. Depletion of *tRF-24* suppresses malignant cell phenotypes in ESCC cells both in vitro and in vivo. Mechanistically, *tRF-24* binds to the Ser28 residue of CELF1, inhibiting AKT1-mediated phosphorylation at this site, which facilitates CELF1 nuclear translocation and subsequent liquid-liquid phase separation (LLPS) formation. These CELF1-enriched nuclear condensates potently regulate the alternative splicing of *BIN1* and *BECN1* pre-mRNAs, generating pro-oncogenic *BIN1-L* and pro-autophagic/mitophagic *BECN1-α* isoforms that collectively enhance tumor malignancy by promoting tumor cell EMT, DNA damage repair, cisplatin resistance and glycolysis. Targeting *tRF-24* with an antagomir significantly suppresses tumor progression in ESCC xenograft models, highlighting its therapeutic potential.

**Conclusions:**

Our findings establish *tRF-24* as a promising therapeutic target in the comprehensive treatment of ESCC.

**Supplementary Information:**

The online version contains supplementary material available at 10.1186/s13046-025-03553-x.

## Introduction

Esophageal cancer is among the most aggressive gastrointestinal malignancies and is the seventh leading cause of cancer-related death worldwide [1]. China has the highest incidence of esophageal cancer globally, with 80% of patients diagnosed with esophageal squamous cell carcinoma (ESCC) [1, 2]. Most patients are diagnosed at advanced or metastatic stages, with a five-year survival rate below 20% [3]. This underscores the urgent need to investigate the molecular mechanisms underlying ESCC development and identify new therapeutic targets.

As an important subclass of non-coding RNAs, tRNA-derived small RNAs (tsRNAs) have become a major research focus due to their diverse regulatory roles in tumor development. tsRNAs are generated through enzymatic cleavage under various cellular conditions and can be primarily categorized into distinct subtypes including 5’ tRFs, 3’ tRFs, i-tRFs, tiRNAs and tRF-1 [4]. tsRNAs play critical roles in malignant progression, including complementarity-based function and RNA-binding protein (RBP)-dependent regulation [4–6]. Typically, tsRNAs associate with Argonaute proteins (AGOs) to form RNA-induced silencing complex (RISC), which is subsequently directed to the partially complementary regions of target mRNAs, predominantly within the 3’ untranslated region (3’UTR). This interaction results in translational repression and mRNA degradation [7, 8]. Alternatively, tsRNAs may regulate transcription regulation [9], post-transcriptional gene expression [10], translation regulation [11] and post-translational modification [12] by directly interacting with RBPs. Despite these mechanistic insights, the functional roles of tsRNAs, especially their regulatory networks in the pathogenesis of ESCC, remain largely unexplored and warrant further investigation.

Liquid-liquid phase separation (LLPS) is a biophysical process in which biomacromolecules condense into membraneless organelles due to physicochemical forces upon reaching a critical phase transition concentration [13]. This process predominantly relies on multivalent interactions, primarily mediated by intrinsically disordered regions (IDRs) of proteins [14]. Dysregulated LLPS has been implicated in the development of human cancers, with increasing evidence showing that RNA molecules are key regulatory factors in phase-separated condensates [15]. For instance, circASH facilitates the LLPS-driven assembly of YBX1/hnRNP complexes, which accelerates the degradation of TPM4 transcripts and suppresses tumor progression in hepatocellular carcinoma [16]. Thus, expanding the repertoire of functional RNAs that regulate LLPS is critical for unraveling their roles in tumor biology.

Here, we identified a tRF, *tRF-24-RPM8309M2S* (*tRF-24*), as a driver of ESCC progression through in-depth analysis of tsRNA-seq data from clinical ESCC specimens. Elevated levels of *tRF-24* are associated with poor patient survival and drive ESCC malignant progression by activating pathways involved in proliferation, invasion, autophagy, mitophagy, EMT, DNA damage repair, cisplatin resistance and glycolysis. Mechanistic studies demonstrate that *tRF-24* directly binds to CUGBP Elav-like family member 1 (CELF1) and inhibits AKT1-mediated phosphorylation of CELF1 at Ser28, leading to increased CELF1 accumulation in the nucleus. Notably, nuclear CELF1 undergoes LLPS, which subsequently enhances its alternative splicing activity. This dysregulation alters the splicing patterns of pre-mRNAs, such as *BIN1* and *BECN1*, thereby contributing to the progression of ESCC.

## Materials and methods

### Study participants and clinical specimens

We analyzed a prospectively maintained cohort of 96 untreated ESCC patients who underwent curative esophagectomy at Qilu Hospital of Shandong University (Jinan, China) between 2017 and 2023 (Supplemental Table 1). Histopathological confirmation was assessed by at least three pathologists, and the staging classification followed the AJCC 8th edition [17]. Ethical review was granted by the Institutional Ethics Committee of Qilu Hospital. Surgically removed tumor specimens and paired adjacent normal tissues were collected during esophagectomy, and were immediately frozen and stored in liquid nitrogen until use. Survival status was determined through medical records, patient families or follow-up calls. Overall survival (OS) was defined as the time from progression to death, and progression-free survival (PFS) was defined as the time from diagnosis to the first recurrence or progression.

#### Analysis of public data

The ESCC tsRNA-seq dataset (GSE207635) was obtained from the Gene Expression Omnibus database for in-depth bioinformatic analysis. It was composed of four ESCC tumors and their paired adjacent normal tissues. A total of 130 tsRNAs were identified in all samples. We chose tsRNAs with Fold Change ≥ 2 and *P* < 0.05 and 76 among them were further selected as significantly upregulated in ESCC compared to normal tissues (Supplemental Table 2). Data processing was carried out using R (v4.3.1), following established pipelines for small RNA-seq analysis.

### Cell culture

The KYSE150 and KYSE30 ESCC cells, along with 293 T cells were purchased from the Cell Bank of Type Culture Collection of the Chinese Academy of Sciences, Shanghai Institute of Biochemistry and Cell Biology. KYSE150 and KYSE30 cells were cultured in RPMI 1640 with 10% FBS, while 293 T cells were cultured in DMEM with 10% FBS. All cells were incubated at 37 °C in a humidified atmosphere with 5% CO2. All cell lines tested negative for Mycoplasma contamination.

### Quantitative real-time PCR (RT-qPCR) and DNA sequencing

Total RNA was extracted from cells or tissues using the SteadyPure Tissue and Cell Small RNA Extraction Kit (Accurate Biology). The rtStarTM tRF and tiRNA Pretreatment Kit (Arraystar) was used to pretreat modifications of RNA samples, and Evo M-MLV RT Kit with gDNA Clean for qPCR II (Accurate Biology) was used to reverse transcription of RNA into cDNA using random primers or specific tRF stem-loop RT primers (synthesized by GenePharma) [18]. Relative RNA levels were measured by quantitative real-time PCR on a LightCycler 480 II system (ROCHE) with SYBR Green reagents (Accurate Biology). *ACTB* and *U6* were used as internal controls for the quantification of mRNAs and tRFs, respectively. Three biological replicates were performed, and expression data were analyzed using the 2^−ΔCt^ method. The cDNAs for tRFs were then cloned into the TA vector and verified by DNA sanger sequencing (GENERAL Biology). All primers are listed in Supplemental Table 3.

### Northern blot analysis

After pre-heated to 90 °C, total RNA (15 µg) isolated from ESCC cells was separated on a 15% denaturing urea-polyacrylamide gel and transferred electrophoretically to Biodyne nylon membranes (Pall Corporation). Membranes were pre-hybridized for 30 min, followed by overnight hybridization at 42 °C with digoxigenin (DIG)-labeled *tRF-24* probes (Supplementary Table 3) in DIG Easy Hyb buffer. After stringent washing, membranes were incubated with anti-digoxigenin-AP (1:10,000), and chemiluminescent signals were detected using an Odyssey infrared imaging system (Li-Cor, Lincoln).

### Plasmid Construction, lentiviral production and stable transfection

Lentivirus-mediated *tRF-24* overexpression or knockdown has been described before [19]. In short, we inserted synthesized *tRF-24* antisense sequence into pLent-U6-shRNA-CMV-luciferase-P2A-puro vector. For overexpression, *tRF-24* sequence was inserted into the same vector containing the miR-30 backbone (WZ Biosciences). The plasmids used in this study are listed in Supplemental Table 4. All plasmids and their insertion sequences were verified by DNA sequencing. For stable transfection, lentiviral plasmids were co-transfected with packaging plasmids into 293 T cells using Lipo3000 transfection reagent (Thermo Fisher Scientific). The produced lentiviruses were mixed with polybrene (Sigma-Aldrich) and added to ESCC cells. Lipo3000 was also used for transient transfection of plasmids, siRNAs or *tRF-24* inhibitors according to the manufacturer’s instructions. The siRNAs and inhibitors used in this study are listed in Supplemental Table 5.

### Analysis of malignant cellular phenotypes

Cell viability was assessed using the CCK-8 assay (Vazyme). For colony formation assays, cell colonies were fixed with methanol and stained with 0.1% crystal violet (Solarbio) before counting. Cell proliferation was measured using an the EdU assay (Abbkine). Cell invasion capacity was assessed using Matrigel-coated 8.0 μm filter membranes. Cells that invaded through the Matrigel (Corning) were stained with crystal violet, counted under a microscope and photographed. Migration ability was tested using the same method, except that the transwell inserts (Costar) were not coated with Matrigel.

### Cell Lysis and Western blot analysis

For Western blot analysis, ESCC cells were lysed in ice-cold 1× RIPA lysis buffer supplemented with the protease and phosphatase inhibitor cocktail (Pierce). The lysates were centrifuged at 12,000 × g for 30 min at 4 °C to collect the supernatant. About 20 µg protein was loaded onto SDS-PAGE gels and transferred to PVDF membranes (Millipore). After blocking with 5% non-fat milk for 1 h, membranes were incubated overnight at 4 °C with primary antibodies, followed by incubation with secondary antibodies (Huabio) for 1 h at room temperature. Immunoblotting bands were visualized with a SuperSignal™ West Pico PLUS Chemiluminescent Substrate (Thermo Scientific). All antibodies used in this study are listed in Supplemental Table 6.

### mCherry-GFP-LC3 reporter assay

Autophagic flux was measured in mCherry-GFP-LC3-transfected ESCC cells, where LC3 is fused to acid-sensitive GFP and pH-stable mCherry. This allows for the differentiation between autophagosomes (yellow puncta: GFP^+^mCherry^+^) and autolysosomes (red puncta: GFP^−^mCherry^+^). Cells were transfected with the mCherry-GFP-LC3 constructs using Lipofectamine 3000, incubated for 48 h under experimental conditions and then scanned using the Opera Phenix^®^ Plus High Content Screening System (PerkinElmer) in confocal mode.

### Mitophagy analysis

Mitophagy was assessed using the MitoTracker Deep Red FM probe (Beyotime Biotechnology) and the GFP-LC3B plasmid (Beyotime Biotechnology). This assay was performed as previously reported [20]. Mitochondrial-autophagosome colocalization was recorded and quantified with the Opera Phenix^®^ Plus High Content Screening System (PerkinElmer) in confocal mode.

### Cell viability assay and colony formation assay

Cells were seeded into 96-well plates at a density of 5 × 10^3^ cells/plate, incubating with cisplatin (Merck) at different concentrations (1–32 µg/mL, diluted with RPMI 1640) for 48 h. Then, cells were treated with CCK-8 reagent for 1 h in the dark. The number of viable cells was determined by measuring absorbance at 450 nm using a microplate reader. For Colony formation assay, ESCC cells were cultured with cisplatin at the indicated concentrations for 3 h. Then, the ESCC cells were harvested and seeded in 6-well plates (500 cells per well). Colonies (≥ 50 cells) were counted by using ImageJ.

### Extracellular acidification rate (ECAR), lactate production and glucose consumption measurements

The Extracellular acidification Rate Assay Kit (BBcellProbe) was used to determine the extracellular acidification rate (ECAR) according to the manufacturer’s instructions. Lactate and glucose concentrations were quantified using a L-Lactate Assay Kit with WST-8 (Beyotime Biotechnology) and a Glucose Uptake Assay Kit with WST-8 (Beyotime Biotechnology), respectively. Absorbance values were measured at the appropriate wavelengths. The obtained results were normalized by the number of cells in each sample in the culture plates, and lactate production and glucose consumption were calculated.

### RNA pulldown assay

RNA pulldown assays were conducted using the Pierce™ Magnetic RNA-Protein Pull-Down Kit (Thermo Scientific). Briefly, biotin-labeled non-targeting control probes, *tRF-24* sense strands or antisense strands (GenePharma) were bound to streptavidin magnetic beads and incubated with cell lysates to construct the RNA-protein binding reaction systems, which was then rotated at 4 °C overnight. The RNA-binding protein complexes were then analyzed by Western blot or further mass spectrometry.

### Mass spectrometric analysis

Proteins captured by the *tRF-24* RNA pulldown assay were quantified and denatured through reduction and alkylation. Trypsin digestion was performed to generate peptides, which were then analyzed by mass spectrometry using a Q-Exactive mass spectrometer (Thermo Scientific). Protein identification was carried out using Proteome Discoverer software. Detailed information for the protein profiles of significant interactions is provided in Supplementary Table 7.

### In vitro Ser28-CELF1 phosphorylation

 In vitro Ser28-CELF1 phosphorylation assay was performed in 20 µL reaction volumes containing kinase buffer (10 mM HEPES, 5 mM DTT, 120 mM KCl, 3 mM MgCl₂, 5% glycerol, 5 mM ATP and 1.25 mM β-glycerophosphate) [12]. Each reaction included 80 ng recombinant CELF1 (Abnova, H00010658-P01), 200 ng active AKT1 kinase (Abcam, ab79792), and adequate amounts of synthesized *tRF-24* or its antisense oligonucleotide. After incubation for 30 min at 30 °C, phosphorylation levels were analyzed by Western blotting using the p-Ser28-CELF1 antibody (Antagene, Phospho-AB2A181), as described previously.

### Cellular phase separation assay

ESCC cell lines stably expressing CELF1-GFP were plated onto PerkinElmer CellCarrier^®^ 96-well plates. Once the cells reached 80% confluency, live-cell imaging was performed using the Opera Phenix^®^ Plus High Content Screening System (PerkinElmer), which was equipped with environmental controls (37 °C, 5% CO₂). GFP-positive puncta (> 0.5 μm in diameter) were identified as phase-separated condensates.

### In vitro phase separation assay

Phase separation assays were performed in reaction buffer containing the specified protein concentrations, supplemented with 10% (w/v) PEG8000, as previously described [16]. Phase-separated droplets were formed in glass-bottom dishes and imaged using a Zeiss LSM980 confocal microscope at 25 °C.

### FRAP assay

FRAP assays were conducted on a Zeiss LSM980 confocal microscope equipped with a 63× oil immersion objective. Photobleaching was achieved using a 488 nm laser at 80% transmission with 10 iterative pulses. Post-bleach imaging captured time-lapse images until fluorescence recovery stabilized. Live-cell experiments were conducted in a humidified imaging chamber maintained at 37 °C with 5% CO₂. Recovery curve analysis was performed using ImageJ software.

### RNA sequencing and alternative splicing analysis

Total RNA was extracted using TRIzol™ Reagent (Thermo Scientific). RNA quality was verified using a NanoDrop™ ND-1000 spectrophotometer and a Bioanalyzer 2100 system (with concentration > 50 ng/µL, RIN ≥ 8.0 and total RNA > 1 µg). Strand-specific libraries were prepared with the TruSeq Stranded mRNA Kit (Illumina, 20020595) and sequenced on an Illumina NovaSeq™ 6000 platform (LC-Bio Technologies) in paired-end mode following standard protocols. Raw sequencing data were filtered for quality to obtain high-quality reads (clean data). Alternative splicing patterns were computationally identified using rMATS with default parameters, including exon skipping, intron retention, alternative 5’or 3’ splice sites and mutually exclusive exons. Sashimi plots are generated with rmats2sashimiplot for visual validation (Supplementary Table 8).

### Hematoxylin and Eosin (H&E) staining

H&E staining was performed following standard protocols. Briefly, after deparaffinization and rehydration, tissue sections were stained with hematoxylin solution (Solarbio, China) for 5 min followed by 5 dips in 1% acid ethanol (1% HCl in 75% ethanol) and then rinsed in distilled water. Cytoplasmic staining was performed using eosin (Solarbio, China) for 3 min. After dehydration through graded ethanol and xylene clearing, the slides were mounted with DPX medium (Sigma) and air-dried. The mounted slides were then imaged using an Olympus VS200 microscope.

### Immunohistochemistry (IHC) staining

Tissue sections were stained using the PV9000 IHC reagent kit (ZSGB Bio, China) following the manufacturer’s instructions. Briefly, formalin-fixed, paraffin-embedded tumor samples were sectioned, and slides were deparaffinized with xylene. Then, the antigen was repaired with EDTA and the catalase blocked with 3% hydrogen peroxide. Subsequently, the sections were blocked with goat serum for 15 min, followed by incubation with the primary antibodies at 4 °C overnight. Finally, the sections were incubated with secondary antibodies, and DAB (ZSGB-Bio, China) and hematoxylin were used to mark the antigen and counterstain the nuclei, respectively.

### RNA fluorescence in situ hybridization (FISH)

RNA FISH was performed using the RNA FISH Kit (Genepharma). Briefly, cells or tissues were fixed in 4% paraformaldehyde (PFA) and hybridized with biotin-labeled *tRF-24* probes (Genepharma) at 37 °C for 16 h in a humidified chamber. Paraffin sections were routinely dewaxed, rehydrated, digested with proteinase K, and hybridized with *tRF-24* probes overnight at 37 °C in the dark. Cell nuclei were counterstained with DAPI. The SA-Biotin System was used to amplify signals, which were detected using the DAPI and Cy3 channels captured by Olympus VS200 microscope.

### Multiplex immunohistochemistry (mIHC)

mIHC was performed using the Treble-Fluorescence Immunohistochemical Mouse/Rabbit Kit (Immunoway). Briefly, after routine dewaxing and rehydration, tumor samples from ESCC patients were subjected to antigen repair and blocking of endogenous peroxidase. After blocking with goat serum, the slices were incubated with the primary antibody. Each staining used a secondary HRP-conjugated antibody with a tyramide-coupled fluorophore. Images were captured using an Olympus VS200 microscope.

### Establishment of mouse xenograft models

Four-week female BALB/c nude mice were purchased from Beijing Vital River Laboratory Animal Technologies and were allowed to acclimate to local conditions for 1 week and were maintained under a 12-hour light/dark cycle with access to sufficient food and water. All animal experiments were carried out in compliance and approved by the Shandong University Specific Pathogen Free (SPF)-Animal Center. For the subcutaneous xenograft model, ESCC cell lines were stably infected with lentiviruses carrying a scramble control, *tRF-24* sense or *tRF-24* antisense sequence. Tumor volumes were measured every three days using a caliper. Maximum tumor size permitted by the ethics committee was not exceeded. AntagotRF-24, designed to target *tRF-24*, consists of a single-strand *tRF-24* with two phosphorothioates at the 5′ end, four phosphorothioates and one cholesterol group at the 3′ end, and one full-length 2′-methoxy modification nucleotide. The intervention was administered in saline at a dose of 40 mg/kg every day for 3 days, followed by every 6 days for an additional 3 times [12].

### Statistics

Statistical analyses were performed according to established biostatistical principles. Group means were compared using Student’s t-test for parametric data. One-way ANOVA and Dunnett’s T3 multiple-comparison tests were applied for multiple group comparisons. Non-parametric analyses of continuous versus dichotomous variables were conducted using the Wilcoxon rank-sum test. Survival outcomes were assessed with univariate log-rank tests and visualized using Kaplan-Meier curves. All computations were performed in SPSS 24.0 (IBM), with statistical significance defined as *P* < 0.05.

### Study approval

Animal handling and experimental procedures were approved by the Animal Care and Animal Experiments Committee of Qilu Hospital, Shandong University. Human tissue samples were obtained with informed consent and approved by the Qilu Hospital Ethics Committee, Shandong University.

### Data Availability

All data needed to evaluate the conclusions are provided herein or in the supplemental material. RNA sequencing data generated in this study have been deposited in the Genome Sequence Archive in BIG Data Center (https://bigd.big.ac.cn/), Beijing Institute of Genomics, Chinese Academy of Sciences, under the accession number: HRA011225. The mass spectrometry proteomics data have been deposited in the ProteomeXchange Consortium (http://proteomecentral.proteomexchange.org) under the dataset identifier PXD063163. All other raw data can be obtained from the corresponding author upon reasonable request.

## Results

### Elevated expression of *tRF-24* is significantly associated with poor survival of ESCC patients

Elevated expression of ***tRF-24*** is significantly associated with poor survival of ESCC patients

**Fig. 1:**
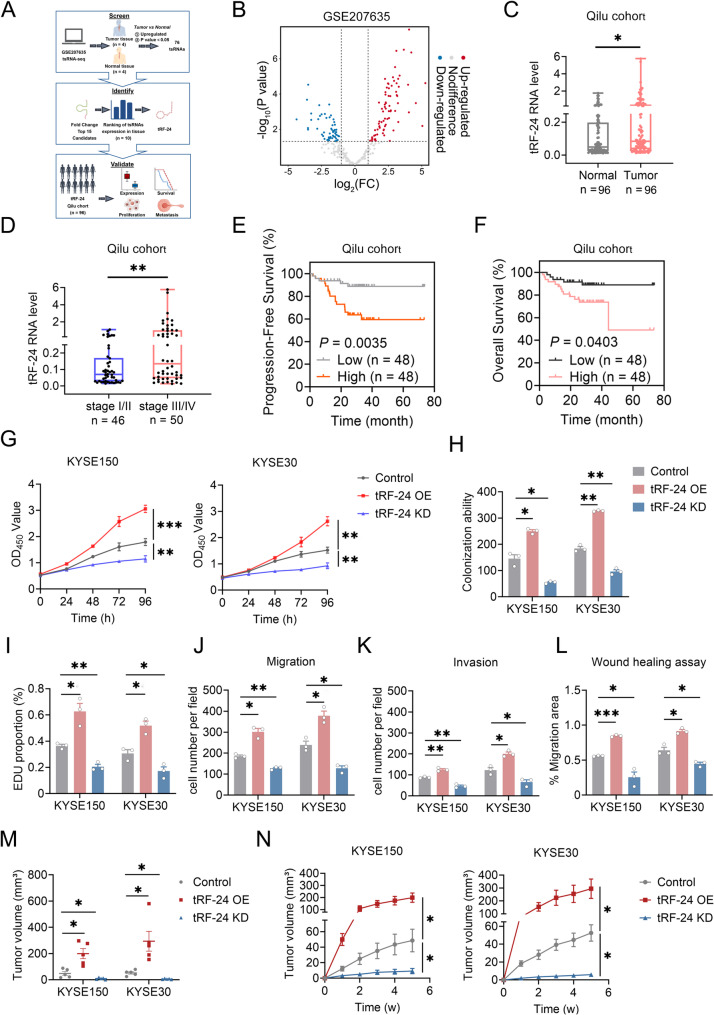
*tRF-24* promotes malignant progression in ESCC (**A**) Screening and validation workflow for tsRNAs with significantly higher expression in ESCC tissues, based on the tsRNA-seq data from the GSE207635 dataset. **B** Volcano plot of tsRNAs differentially expressed between ESCC tissues and paired normal tissues from the GSE207635. Red and blue dots indicate tsRNAs with *P* < 0.05 and |Fold Change| >2, whereas gray dots indicate *P* ≥ 0.05. **C**, **D** *tRF-24* levels in paired ESCC and adjacent normal tissues (**C**) and in stage III/IV tumors and stage I/II tumors (**D**) from the Qilu cohort (*n* = 96). Data are shown as box plots; the lines in the middle of the box indicate the median, and the upper and lower lines indicate the 25th and 75th percentiles. *P < 0.05, **​*P* < 0.01 by the Wilcoxon rank-sum test. **E, F** Kaplan-Meier survival analysis of patients’ progression-free survival (PFS) (**E**) and overall survival (OS) (**F**) stratified by the median expression of *tRF-24*. *P* values were determined by log-rank test. **G-L** Functional effects of *tRF-24* overexpression (OE) or knockdown (KD) on ESCC malignant phenotypes. **G** CCK-8 assay for cell viability. **H** Colony formation assay. **I** EdU proliferation assay. **J, K** Transwell migration and invasion assay. **L** Wound healing assay. **M**,** N** Endpoint tumor weights (**M**) and growth curves (**N**) of subcutaneous xenografts in BALB/c nude mice. Data in (**G-N**) are shown as mean ± SEM from 3 independent experiments. *P < 0.05, **​P < 0.01, ***​*P* < 0.001 by one-way ANOVA with Dunnett’s T3 multiple-comparison test

We began with tsRNA-seq data from ESCC samples in the Gene Expression Omnibus (GEO ID: GSE207635), which includes four pairs of matched tumor and adjacent normal tissues, to identify significantly upregulated tRFs. Among the 76 tsRNAs significantly upregulated in ESCC tumors, the top fifteen were selected for further validation using quantitative real-time PCR (qRT-PCR) in a cohort of ESCC patients from Qilu hospital (Fig. 1A-B, Supplementary Fig. 1 A, Supplementary Table 2). Pilot validation in 10 matched ESCC tumor and adjacent normal tissues revealed that *tRF-24* was the most upregulated in neoplastic lesions compared to normal counterparts (Supplementary Fig. 1 A). Then we verified the expression of *tRF-24* in a larger cohort of 96 ESCC patients and found that its levels were significantly higher in ESCC tumors than in adjacent normal tissues (Fig. 1C). Additionally, *tRF-24* levels were higher in advanced-stage tumors (stages III/IV) than in early-stage tumors (stages I/II) (Fig. 1D). Kaplan-Meier analysis showed that ESCC patients with high *tRF-24* levels had significantly shorter progression-free and overall survival compared to those with low *tRF-24* levels in the Qilu cohort (Fig. 1E-F). Our findings indicate that *tRF-24* promotes ESCC progression and correlates with disease stage. These results suggest its potential as both a prognostic biomarker and a therapeutic target for ESCC progression.

It has been reported that *tRF-24* is a 24-nt 5’-tRF generated by anticodon loop processing of mature *tRNA*^*LeuAAG/TAG*^ (Supplementary Fig. 1B) [21]. Specific amplification using stem-loop primers was validated by sequencing, confirming that it corresponds to mature *tRF-24* (Supplementary Fig. 1 C). We quantified *tRF-24* expression across six established ESCC cell lines. KYSE150 and KYSE30 exhibited intermediate expression levels and were selected for subsequent functional characterization (Supplementary Fig. 1D). Northern blot analysis validated *tRF-24* expression in KYSE150 and KYSE30 ESCC cell lines (Supplementary Fig. 1E). To delineate the subcellular distribution of *tRF-24*, we performed nucleocytoplasmic fractionation followed by qRT-PCR quantification and found that *tRF-24* was preferentially enriched in the cytosol (Supplementary Fig. 1F).

### *tRF-24* promotes malignant progression in ESCC

We next examined the effects of *tRF-24* on ESCC cell phenotypes by altering its levels in cells (Supplementary Fig. 2A), but changing *tRF-24* levels did not affect the abundance of cellular mature *tRNA*^*LeuAAG/TAG*^ (Supplementary Fig. 2B). Functional studies demonstrated that *tRF-24* plays an important role in ESCC progression. Overexpression of *tRF-24* enhanced tumor cell proliferation, migration and invasion, while knockdown reversed these effects (Fig. 1G-L, Supplementary Fig. 2C-F). Given that EMT is crucial for enhancing migration and invasion, we next investigated whether the effects of *tRF-24* on these processes are associated to EMT progression [22–25]. The results revealed that the *tRF-24* overexpression led to decreased expression of E-cadherin but increased expression of N-cadherin, Vimentin and SNAIL. Conversely, *tRF-24* knockdown had the opposite effect (Supplementary Fig. 2G).

To investigate the in vivo role of *tRF-24*, we generated KYSE150 and KYSE30 cell lines with either *tRF-24* overexpression or knockdown and established corresponding subcutaneous xenograft models. Consistent with in vitro findings, overexpression of *tRF-24* markedly accelerated tumor growth, while knockdown of *tRF-24* suppressed tumor growth compared to control (Fig. 1M-N, Supplementary Fig. 2H-I). Collectively, these results establish *tRF-24* as a major driver of ESCC malignancy.

### *tRF-24* facilitates autophagy and mitophagy in ESCC

**Fig. 2:**
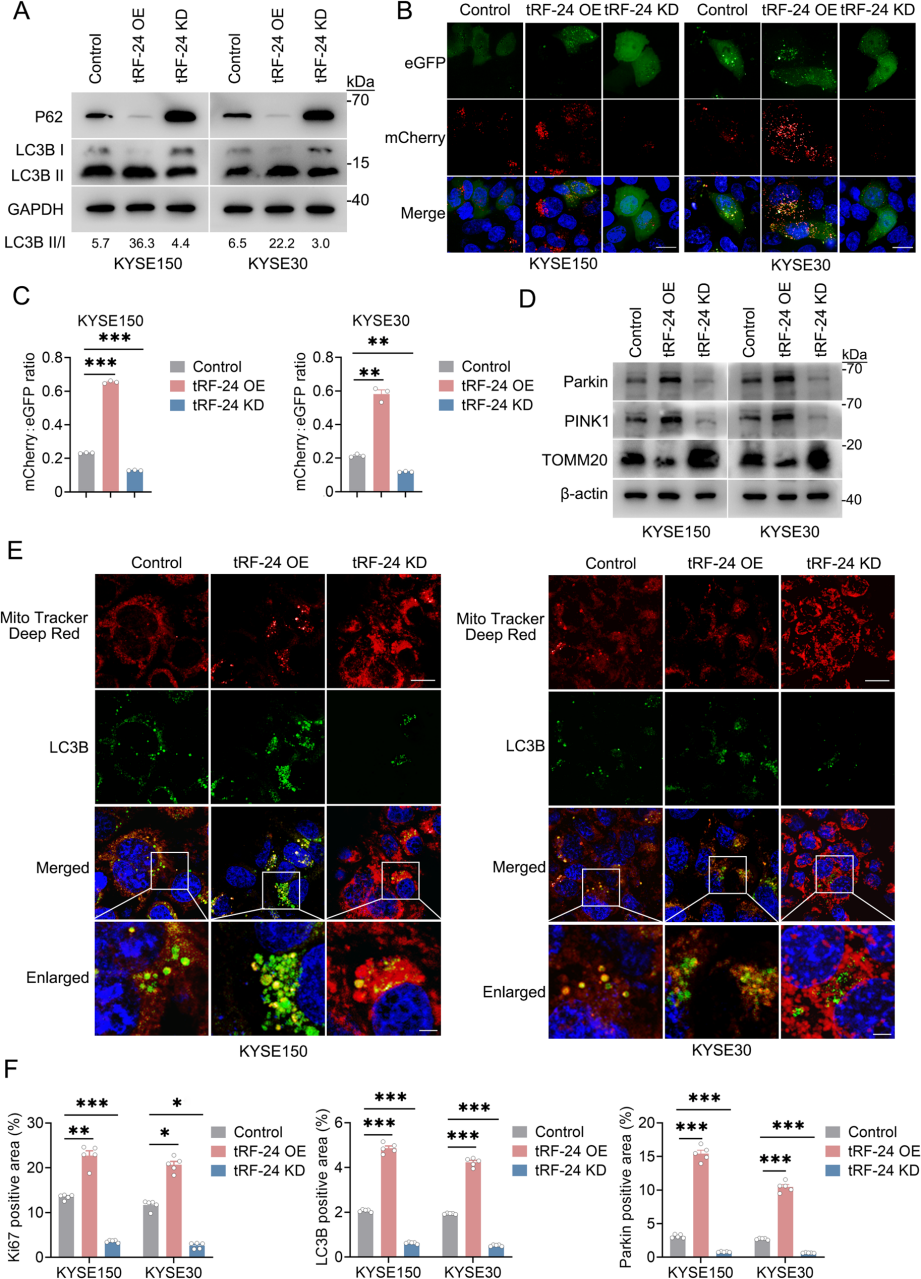
*tRF-24* promotes autophagy and mitophagy in ESCC (**A**) Western blot analysis of LC3-I to LC3-II conversion and p62 expression in ESCC cells with *tRF-24* overexpression (OE) or knockdown (KD). GAPDH serves as a loading control. **B** Autophagic flux assessed using the mCherry-GFP-LC3 reporter assay. Yellow puncta (mCherry^+^/GFP^+^) represent autophagosomes, and red puncta (mCherry^+^/GFP^−^) represent autolysosomes. Scale bars: 20 μm. **C** Quantitative analysis of the mCherry/GFP fluorescence ratio reflects autophagic flux. Higher ratios indicate efficient lysosomal acidification and autolysosome formation, while lower ratios reflect autophagosome accumulation. **D** Western blot analysis showing Parkin, PINK1 and TOMM20 levels under distinct *tRF-24* expression conditions. β-actin is used as a loading control. **E** Colocalization analysis of MitoTracker Deep Red (mitochondria) and GFP-LC3 (autophagosomes) following changes in *tRF-24* expression. Scale bars: 20 μm for overview and 5 μm for inset. **F** Quantitative analysis of Ki67, LC3B and Parkin expression in subcutaneous xenografts (*n* = 5). Positivity was determined by threshold-based segmentation of IHC staining. Data in (**C**,** F**) are presented as mean ± SEM from 3 independent experiments. *P < 0.05, **​*P* < 0.01, ***​*P* < 0.001 by one-way ANOVA with Dunnett’s T3 multiple-comparison test

Autophagy facilitates the clearance of damaged cellular components through catabolic recycling, promoting progression in established tumors [26]. Increasing evidence shows that autophagy plays an important role in oncogenic processes, including maintaining cell viability [27], enhancing migratory invasion [28], acquiring anoikis resistance [29], and promoting EMT progression [30]. Mitophagy, a form of selective autophagy, specifically removes damaged mitochondria. This process protects cells by reducing apoptosis and supporting tumor cell survival under stress conditions, such as nutrient deprivation and hypoxia [31]. Therefore, we investigated whether *tRF-24* exerted oncogenic effects through autophagy and mitophagy in ESCC. Our data showed that overexpression of *tRF-24* significantly increased the LC3-II/I ratio and decreased p62 levels, while silencing of *tRF-24* had the opposite effects (Fig. 2A). To monitor autophagic flux, we used the GFP-mCherry-LC3 reporter system. GFP fluorescence is quenched in acidic environments, while mCherry fluorescence remains stable across pH changes, allowing precise differentiation between autophagosomes (GFP^+^/mCherry^+^) and autolysosomes (GFP^−^/mCherry^+^). Overexpression of *tRF-24* led to autophagosome-lysosome accumulation, which was reversed by its knockdown (Fig. 2B-C).

Furthermore, to determine the roles of *tRF-24* in mitophagy regulation in ESCC, we analyzed the mitochondrial marker TOMM20, as well as mitophagy regulators PINK1 and Parkin, after altering *tRF-24* levels. The results demonstrated that the levels of PINK1 and Parkin exhibited a similar pattern to *tRF-24*, with increased levels following *tRF-24* overexpression and decreased upon *tRF-24* silencing (Fig. 2D). Mitochondrial GFP-LC3 co-localization significantly increased with *tRF-24* overexpression compared to the control groups, while *tRF-24* knockdown showed the opposite pattern (Fig. 2E).

Xenograft mouse models replicated the in vitro pro-autophagic/mitophagic effects mediated by *tRF-24*. Immunohistochemical (IHC) analysis of subcutaneous xenograft tissues demonstrated significantly increased expression of Ki67, LC3 and Parkin in tumors with *tRF-24* overexpression. In contrast, silencing *tRF-24* led to a marked reduction in these biomarkers (Fig. 2F, Supplementary Fig. 3). Taken together, *tRF-24* overexpression activates autophagy and mitophagy, highlighting its critical roles in ESCC survival.

### *tRF-24* disrupts CELF1 phosphorylation and promotes nuclear translocation

**Fig. 3:**
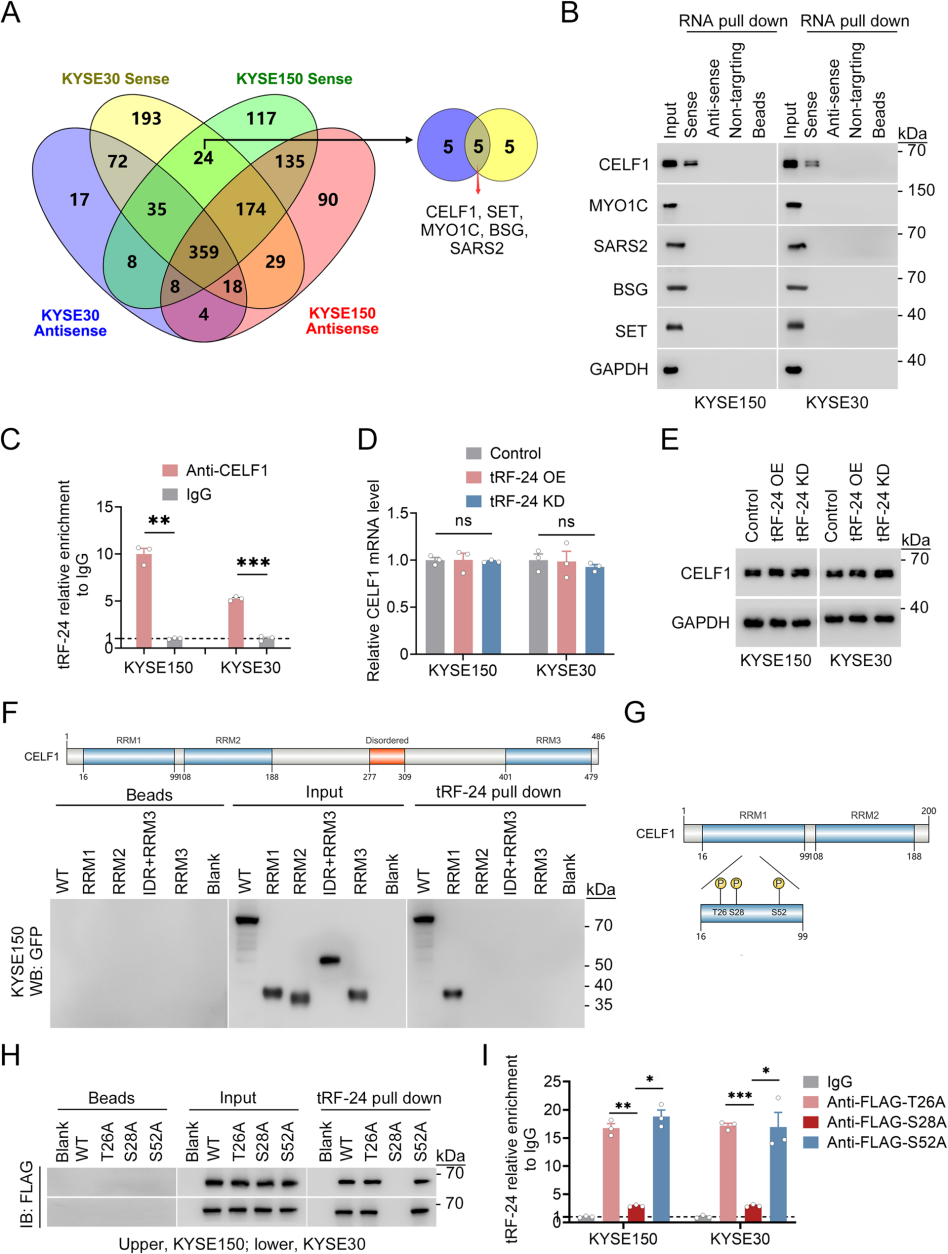
*tRF-24* interacts with CELF1 at Ser28 phosphorylation site (**A**) Venn diagram showing proteins identified by RNA pull-down with *tRF-24* sense or antisense probes, followed by LC-MS analysis. Specific *tRF-24* binding candidates were defined as proteins enriched in the sense group but absent in the antisense group. **B** Validation of seven candidate *tRF-24* binding proteins using RNA pull-down followed by immunoblotting. **C** RIP-qPCR analysis of CELF1 binding to *tRF-24* in ESCC cells. IgG was used as a negative control. Data are shown as mean ± SEM from 3 independent experiments. ** *P* < 0.01, *** *P* < 0.001 by Student’s *t* test. **D** *tRF-24* overexpression (OE) or knockdown (KD) does not affect *CELF1* mRNA levels. Data are shown as mean ± SEM from 3 independent experiments. ns, no significance, by one-way ANOVA with Dunnett’s T3 multiple-comparison test. **E **Western blot analysis showing that CELF1 protein levels remain unchanged after *tRF-24* overexpression (OE) or knockdown (KD). **F** Truncation mapping of the *tRF-24*–CELF1 binding domain. The schematic diagram shows the GFP-tagged CELF1 protein domain structure (*Top*). Western blot analysis shows GFP-tagged full-length (WT) CELF1 and its truncated forms pulled down by *tRF-24* (*Bottom*). **G** Phosphorylation sites in the RRM1 domain. **H** Immunoblot analysis showing FLAG-tagged full-length CELF1 (WT) and mutants (T26A, T28A, S52A) pulled down by *tRF-24*. **I** RIP assays with an antibody against FLAG-tagged full-length CELF1 (WT) and mutants (T26A, T28A, S52A). Data represent enrichment (mean ± SEM) relative to input from three independent experiments. IgG was used as a negative control. *P < 0.05, **​*P* < 0.01, ***​*P* < 0.001 by one-way ANOVA with Dunnett’s T3 multiple-comparison test

**Fig. 4:**
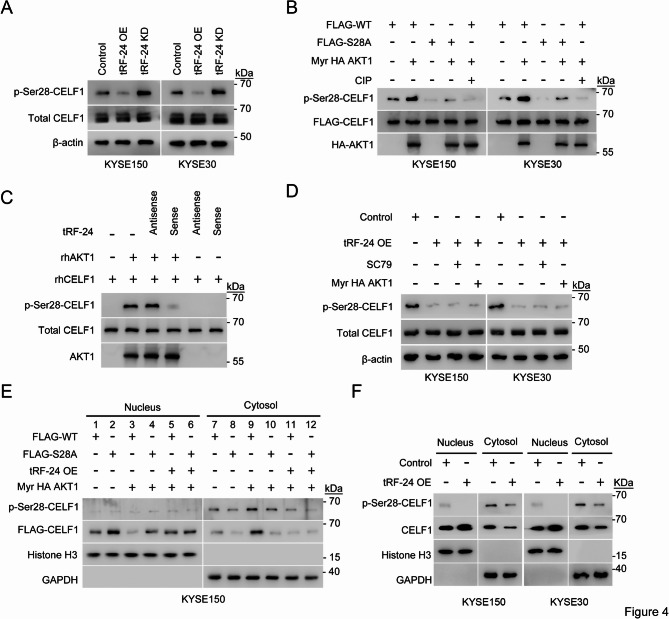
*tRF-24* inhibits AKT1-mediated phosphorylation of CELF1 at Ser28 (**A**) Western blot analysis of p-Ser28-CELF1 in ESCC cells with *tRF-24* overexpression (OE) or knockdown (KD). **B** Myr-HA-AKT1 phosphorylated FLAG-CELF1 at Ser28 in ESCC cells. Calf intestinal alkaline phosphatase (CIP) was used for 1 h at 37 °C. **C** Effects of *tRF-24* sense or antisense on in vitro phosphorylation of CELF1 at Ser28 by recombinant human AKT1 (rhAKT1). CELF1 was used as a loading control. **D** Effects of the AKT activator SC79 or Myr-HA-AKT1 on the phosphorylation of CELF1 in the presence of *tRF-24*. SC79 (4 µg/mL) was added and incubated for 30 min. **E** Immunoblot analysis of p-Ser28-CELF1 and total CELF1 localization in fractionated ESCC cells transfected with FLAG-CELF1 (wild-type, WT: lanes 1, 3, 5, 7, 9, 11) or FLAG-S28A mutant CELF1 (FLAG-S28A: lanes 2, 4, 6, 8, 10, 12), in combination with *tRF-24* (lanes 5, 6, 11, 12) and Myr-HA-AKT1 (lanes 3, 4, 5, 6, 9, 10, 11, 12). **F** Immunoblot analysis of p-Ser28-CELF1 and total CELF1 localization in fractionated ESCC cells stably overexpressing *tRF-24*. Histone H3 was used as a nuclear control and GAPDH as a cytoplasmic control

To explore the oncogenic mechanism of *tRF-24* in ESCC, we conducted RNA pull-down assays using biotinylated *tRF-24* or its antisense control, followed by mass spectrometry (MS) analysis. We identified 24 proteins in KYSE150 and KYSE30 cells that specifically bind to *tRF-24*, compared to the antisense control (Fig. 3A, Supplementary Table 7). We then validated the five most abundant candidates using RNA pull-down assays, demonstrating a specific interaction between *tRF-24* and CELF1 (Fig. 3B), an RNA-binding protein involved in regulating alternative splicing and stabilizing mRNAs [32, 33]. Consistent with these findings, RNA immunoprecipitation (RIP) using a CELF1 antibody showed a physical interaction between *tRF-24* and CELF1 in ESCC cells (Fig. 3C). Notably, neither CELF1 mRNA nor protein levels exhibited significant changes following *tRF-24* overexpression or knockdown, suggesting that *tRF-24* might regulate CELF1 activity through post-translational mechanisms (Fig. 3D, E). To identify the *tRF-24* binding domains in CELF1, we constructed GFP-tagged truncations of CELF1, as FLAG-tagged RNA recognition motifs (RRM1 and RRM2) were unstable. Domain truncation analysis showed that the RRM1 domain of CELF1 mediates *tRF-24* binding (Fig. 3F). Three phosphorylation sites (Thr26, Ser28, Ser52) have been identified in the CELF1 RRM1 domain (Fig. 3G) [34]. Phosphorylation at Ser28 is associated with changes in the nucleocytoplasmic distribution of the protein [35]. To investigate whether *tRF-24* can affect Ser28 phosphorylation, RNA pull-down assays were performed using ESCC cell lysates expressing FLAG-tagged wild-type CELF1 or site-directed mutants (Thr26Ala, Ser28Ala, Ser52Ala). The results showed that the Ser28Ala mutation specifically disrupted *tRF-24* binding to CELF1, while the Thr26Ala and Ser52Ala mutations did not affect the binding (Fig. 3H). RIP analysis confirmed the specific interaction between *tRF-24* and CELF1 Ser28, indicating that Ser28 is a critical residue of CELF1 for *tRF-24* binding (Fig. 3I). Western blot analysis using an antibody against phosphorylated CELF1 at Ser28 (p-Ser28-CELF1) was then performed to assess whether CELF1 phosphorylation changes in cells with altered *tRF-24* levels. The data demonstrated that *tRF-24* overexpression repressed CELF1 Ser28 phosphorylation, while silencing *tRF-24* promoted this process (Fig. 4A). To confirm AKT1-mediated phosphorylation at CELF1 Ser28 [36] and investigate the regulatory roles of *tRF-24*, we established ESCC cells stably expressing a constitutively active AKT1 mutant (Myr-HA-AKT1). In these cells, the levels of phosphorylated wild-type CELF1 were significantly increased, while the phospho-signal for Ser28Ala-CELF1 mutant remained undetectable. Phosphatase treatment reduced p-Ser28-CELF1 levels significantly (Fig. 4B). Further in vitro validation demonstrated that *tRF-24* blocked CELF1 phosphorylation, while the antisense RNA exhibited no such effects (Fig. 4C). Notably, these inhibitory effects of *tRF-24* persisted even after treatment with the AKT pathway agonist SC79 or overexpression of constitutively active AKT1 in ESCC cells (Fig. 4D). Collectively, these findings demonstrate that *tRF-24* suppresses AKT1-mediated phosphorylation of CELF1.

Phosphorylation of CELF1 at Ser28 regulates its nucleocytoplasmic shuttling, prompting investigation into the roles of *tRF-24* in this process. Transfection of ESCC cells with FLAG-tagged phospho-mimetic (Ser28) or phospho-null (Ala28) CELF1 constructs revealed distinct subcellular localization patterns. Co-expression of Myr-HA-AKT1 with wild-type CELF1 led to the movement of CELF1 to the cytoplasm (Fig. 4E, lanes 3, 9). However, pretreatment with *tRF-24* mimics retained wild-type CELF1 in the nucleus, even with AKT1 co-expression (Fig. 4E, lanes 5, 11). In contrast, the Ser28Ala-CELF1 mutant exhibited persistent nuclear localization, unaffected by AKT1 activation or *tRF-24* levels alteration (Fig. 4E, lanes 2, 4, 6, 8, 10 and 12). Consistent with these results, ESCC cells overexpressing *tRF-24* showed similar findings (Fig. 4F). In summary, our findings show that AKT1-mediated phosphorylation regulates CELF1 nucleocytoplasmic shuttling, while *tRF-24* selectively disrupts this process by reducing Ser28 phosphorylation of CELF1.

### CELF1 undergoes liquid-liquid phase separation in the nuclear compartments

LLPS is widely recognized as the mechanism behind the formation of biomolecular condensates, which are associated with specific molecular aggregation processes [37]. Our data described above showed that *tRF-24* mediates the nuclear enrichment of CELF1 (Fig. 4E-F). We then hypothesized that this redistribution increased local concentrations, which may further promote CELF1 phase separation in the nucleus. In our experiments, ectopic expression of GFP-CELF1 consistently formed nuclear punctate structures (Supplementary Fig. 4 A), prompting us to examine whether these condensates exhibited typical LLPS characteristics.

**Fig. 5:**
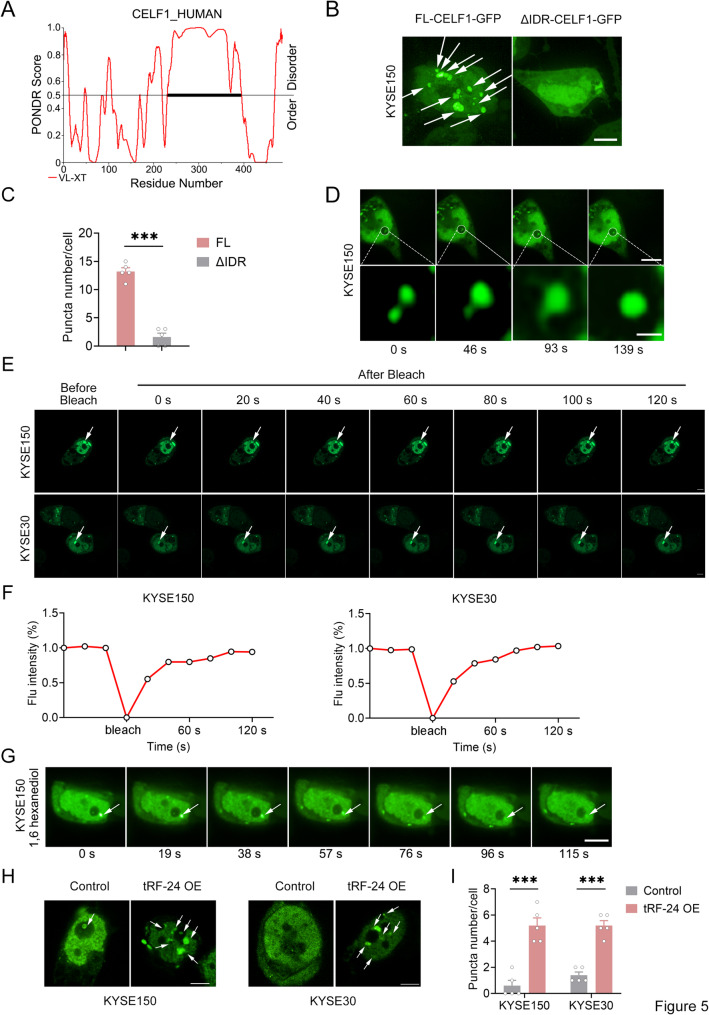
CELF1 undergoes LLPS in the nucleus. **A** Prediction of the intrinsically disordered region (IDR) of CELF1. **B**, **C** KYSE150 cells transfected with FL-CELF1-GFP or △IDR-CELF1-GFP were analyzed using the Opera Phenix™ High Content Screening System. Representative images (**B**) and quantification of CELF1-GFP puncta per cell (**C**). Scale bars: 10 μm. **D** Time-series fluorescence microscopy analysis of CELF1-GFP puncta in ESCC cells (*Upper panel*). Scale bars: 10 μm. The zoomed-in view of two fusing puncta is shown in the *Bottom panel*. Scale bars: 1 μm. **E–F** Representative micrographs of CELF1-GFP puncta before and after photobleaching in ESCC cells (**E**). Scale bars: 5 μm. Quantification of fluorescence intensity recovery in the bleached region of CELF1 puncta (**F**). **G** The effects of 5% 1,6-hexanediol on CELF1 droplets *in vivo.* Scale bars: 20 μm. (**H–I**) Representative fluorescent images of CELF1-GFP in *tRF-24* overexpressed or control ESCC cells (**H**). Scale bars, 20 μm. Quantification of CELF1-GFP puncta numbers (**I**). Data in (**C**,** I**) are shown as mean ± SEM from 3 independent experiments. ***P < 0.001, by Student’s *t* test

There is growing consensus that IDRs are critical drivers of LLPS [38]. Structural analysis of CELF1 showed a prominent IDR in its primary structure (Fig. 5A). To examine the phase separation ability of CELF1 in ESCC, we expressed GFP-tagged full-length CELF1 (GFP-FL-CELF1) or a mutant lacking the IDR (GFP-ΔIDR-CELF1) in ESCC cells. Confocal microscopy revealed distinct nuclear puncta in GFP-FL-CELF1 cells, while GFP-ΔIDR-CELF1 cells exhibited diffuse nuclear staining (Fig. 5B-C). Live imaging showed dynamic coalescence of CELF1 puncta in ESCC cells, where two separate puncta merged into a larger structure (Fig. 5D). Fluorescence recovery after photobleaching (FRAP) analysis confirmed the presence of LLPS in CELF1 condensates within the nucleus (Fig. 5E-F). Treatment with the LLPS inhibitor 1,6-hexanediol abolished CELF1 nuclear puncta formation (Fig. 5G). Our multiscale analysis above provided direct evidence that CELF1 undergoes phase separation in ESCC cells.

We further performed in vitro biomolecular condensation assays using PEG8000, a validated inducer of molecular crowding, after purified GFP-FL-CELF1 and GFP-ΔIDR-CELF1 proteins. Our experiments demonstrated that GFP- FL-CELF1 protein formed time-dependent LLPS in vitro, while GFP-ΔIDR-CELF1 protein did not form such structures under the same conditions (Supplementary Fig. 5B-C). Notably, treatment with 1,6-hexanediol completely disrupted the LLPS droplets (Supplementary Fig. 5D). To confirm the liquid-like behavior of recombinant CELF1 condensates in vitro, we conducted FRAP analysis using purified GFP-FL-CELF1 protein. Quantitative tracking showed almost complete fluorescence recovery in GFP-tagged droplets after photobleaching (Supplementary Fig. 5E-F). Additionally, orthogonal validation experiments demonstrated that elevated CELF1 concentrations and optimized NaCl conditions promoted LLPS (Supplementary Fig. 5G). Overall, these results show that CELF1 induces LLPS both in vivo and in vitro, and that the IDR is crucial for LLPS formation.

Previous studies have reported that long non-coding RNAs (lncRNAs) were essential for the LLPS process [39]. However, the roles of tsRNAs in the formation of these biomolecular condensates remain unexplored. Our results demonstrated that *tRF-24* promoted the nuclear translocation of CELF1 by inhibiting Ser28 phosphorylation, which leading its accumulation in the nucleus (Fig. 4). This observation prompted further investigation into the role of *tRF-24* in regulating CELF1-formed LLPS. As expected, overexpression of *tRF-24* caused significant nuclear enrichment of CELF1 condensates (Fig. 5H-I), suggesting that *tRF-24* modulates CELF1 LLPS by altering subcellular compartmentalization.

### *tRF-24* regulates CELF1-mediated alternative splicing of *BIN1* and *BECN1* pre-mRNAs

**Fig. 6:**
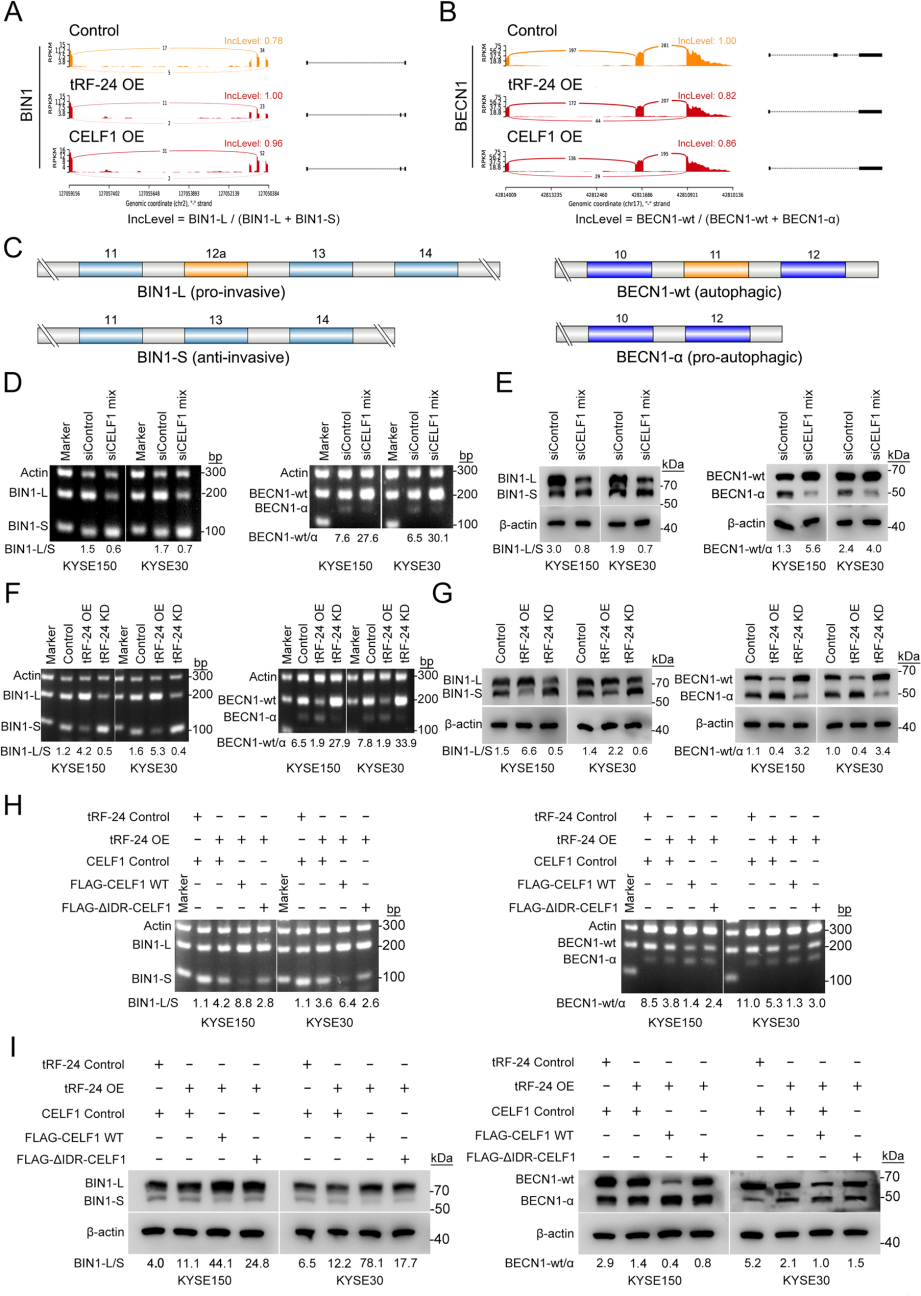
tRF-24 promotes alternative splicing of the ***BIN1*** and ***BECN1*** mediated by CELF1 (**A**,** B**) Increased inclusion of *BIN1* exon 12a (**A**) and decreased inclusion of *BECN1* exon 11 (**B**) in mature mRNA of KYSE150 cells with *tRF-24* or CELF1 overexpression. **C** Diagrams of the two splice variants of *BIN1* (*left*) and *BECN1* (*right*) mRNA and their functions. **D**,** E** Semiquantitative RT-PCR analysis showing the relative mRNA ratios of *BIN1-L/S* (*left*) and *BECN1-wt/α* (*right*) after *CELF1* silencing (**D**). Western blot analysis of corresponding protein ratios (**E**). **F**,** G** Effects of changes in *tRF-24* expression on the alternative splicing of *BIN1-L/S* (left) and *BECN1-wt/α* (right) mRNA (**F**) and protein levels (**G**). **H**, **I** Semiquantitative RT-PCR analysis of *BIN1-L/S* (*left*) and *BECN1-wt/α* (*right*) mRNA splicing after transfection of FLAG-WT-CELF1 and FLAG-△IDR-CELF1 (**H**). Western blot analysis of the corresponding protein ratios (**I**)

Nuclear CELF1 is well known as a key regulator of alternative splicing [40]. To explore the role of the *tRF-24*-CELF1 axis in ESCC tumorigenesis through alternative splicing regulation, we performed RNA sequencing (RNA-seq) analysis of KYSE150 cells with *tRF-24* or CELF1 overexpression, as well as negative control. This study aimed to identify alternative splicing events regulated by *tRF-24* and CELF1 in ESCC pathogenesis. RNA-seq analysis revealed that overexpression of *tRF-24* or CELF1 in ESCC cells resulted in significant changes in alternative splicing patterns. Multivariate analysis of transcript splicing showed that exon skipping was the most common alternative splicing event (Supplementary Fig. 5 A). Bioinformatics analysis identified 250 splicing-regulated genes (FDR < 0.05, |IncLevel Difference| >0.1) that responded to both *tRF-24* and CELF1 overexpression. Among these genes, *BIN1* acts as a tumor suppressor and is dysregulated in various human cancers [41–43]. Notably, tumor-associated alternative splicing produces the pro-oncogenic *BIN1-L* isoform while reducing the tumor-suppressive *BIN1-S* variant [44]. The *BECN1* gene encodes Beclin-1, a key protein acting as a major regulator of both autophagy [45] and mitophagy [46]. Recent studies have demonstrated that alternative splicing of *BECN1* generates the *BECN1-α* isoform, which induces excessive activation of both autophagy and mitophagy pathways [47]. RNA sequencing analysis showed that CELF1 overexpression regulated alternative splicing events in various ways. Specifically, it impaired *BIN1* splicing efficiency, promoting exon 12a retention and generating the elongated *BIN1-L* isoform. In contrast, it enhanced *BECN1* splicing efficiency, leading to the exclusion of exon 11 and producing the truncated *BECN1-α* isoform (Fig. 6A-C). Further validation demonstrated that silencing CELF1 significantly decreased the *BIN1-L/S* ratio and increased the *BECN1-wt/α* ratio compared to control cells (Fig. 6D, Supplementary Fig. 5B). These splicing changes were also reflected in protein expression (Fig. 6E). *tRF-24* knockdown produced similar effects to CELF1 silencing, while *tRF-24* overexpression induced the *BIN1-L* and *BECN1-α* isoforms (Fig. 6F-G). Given the oncogenic roles of *BIN1* and *BECN1*, and their regulation by CELF1-mediated splicing, we conducted rescue experiments in ESCC cells to validate the function of the *tRF-24*-CELF1-*BIN1*/*BECN1* regulatory axis. Our data show that CELF1 downregulation mitigated the increased cell proliferation, migration, invasion, EMT, autophagy and mitophagy observed in *tRF-24*-overexpressing cells (Supplementary Fig. 5C-E, Supplementary Fig. 6A-E). CELF1 knockdown reversed the *tRF-24*-induced imbalance in the *BIN1-L/S* and *BECN1-wt/α* ratios, as well as the corresponding protein isoform expression patterns (Supplementary Fig. 7A-B). Our results demonstrate that *tRF-24* regulates CELF1-mediated alternative splicing of *BIN1*/*BECN1* pre-mRNAs.

It is well established that different BIN1 isoforms play distinct roles in DNA damage repair, cisplatin resistance and energy metabolism [48, 49]. BIN1-S inactivates ataxia telangiectasia–mutated (ATM) serine/threonine kinase by binding to E2F1, thereby suppressing double-strand DNA damage repair and cisplatin resistance. In contrast, BIN1-L, which does not interact with E2F1, counteracts these effects [48]. Notably, BECN1 also promote DNA damage repair and cisplatin resistance through autophagy, suggesting that alterations in BECN1 splicing could influence this process [50–52]. In our experiments, *tRF-24* overexpression enhanced DNA damage repair and cisplatin resistance in ESCC cells, while its downregulation had the opposite effect (Supplementary Figs. 8 and 9). Furthermore, rescue experiments confirmed that these effects are mediated by the *tRF-24-*CELF1*-BIN1/BECN1* regulatory axis (Supplementary Figs. 10 and 11). Additionally, BIN1-S specifically interacts with c-myc to inhibit its transcription, thereby suppressing glycolysis, whereas BIN1-L lacks this ability [44, 49, 53]. Consistently, our results showed that *tRF-24* overexpression promotes glycolysis, whereas its knockdown had the opposite effect, and this process was mediated by CELF1 (Supplementary Figs. 12 and 13). Taken together, our findings demonstrate that *tRF-24* modulates CELF1-mediated alternative splicing of *BIN1* and *BECN1*, promoting DNA damage repair, cisplatin resistance and glycolytic metabolism in ESCC.

Emerging evidence shows that proteins involved in LLPS to form biomolecular condensates serve multifunctional roles as biochemical reaction centers, signaling hubs and structural scaffolds. Membraneless organelles are essential for coordinating key cellular processes, such as alternative splicing [54, 55], DNA damage response [56] and transcriptional regulation [57]. Therefore, we hypothesize that *tRF-24* regulates the alternative splicing of *BIN1* and *BECN1* pre-mRNAs by modulating the LLPS dynamics of CELF1. Consistent with expectation, full-length CELF1 expression significantly enhanced the splicing patterns of *BIN1* and *BECN1* induced by *tRF-24* overexpression. In contrast, the ΔIDR variant showed no detectable effects on these splicing events (Fig. 6H–I). Therefore, these findings demonstrate that the role of CELF1 in regulating alternative splicing depends on LLPS, mediated by its IDR.

### Targeting *tRF-24* exhibits promising preclinical therapeutic efficacy in ESCC

**Fig. 7:**
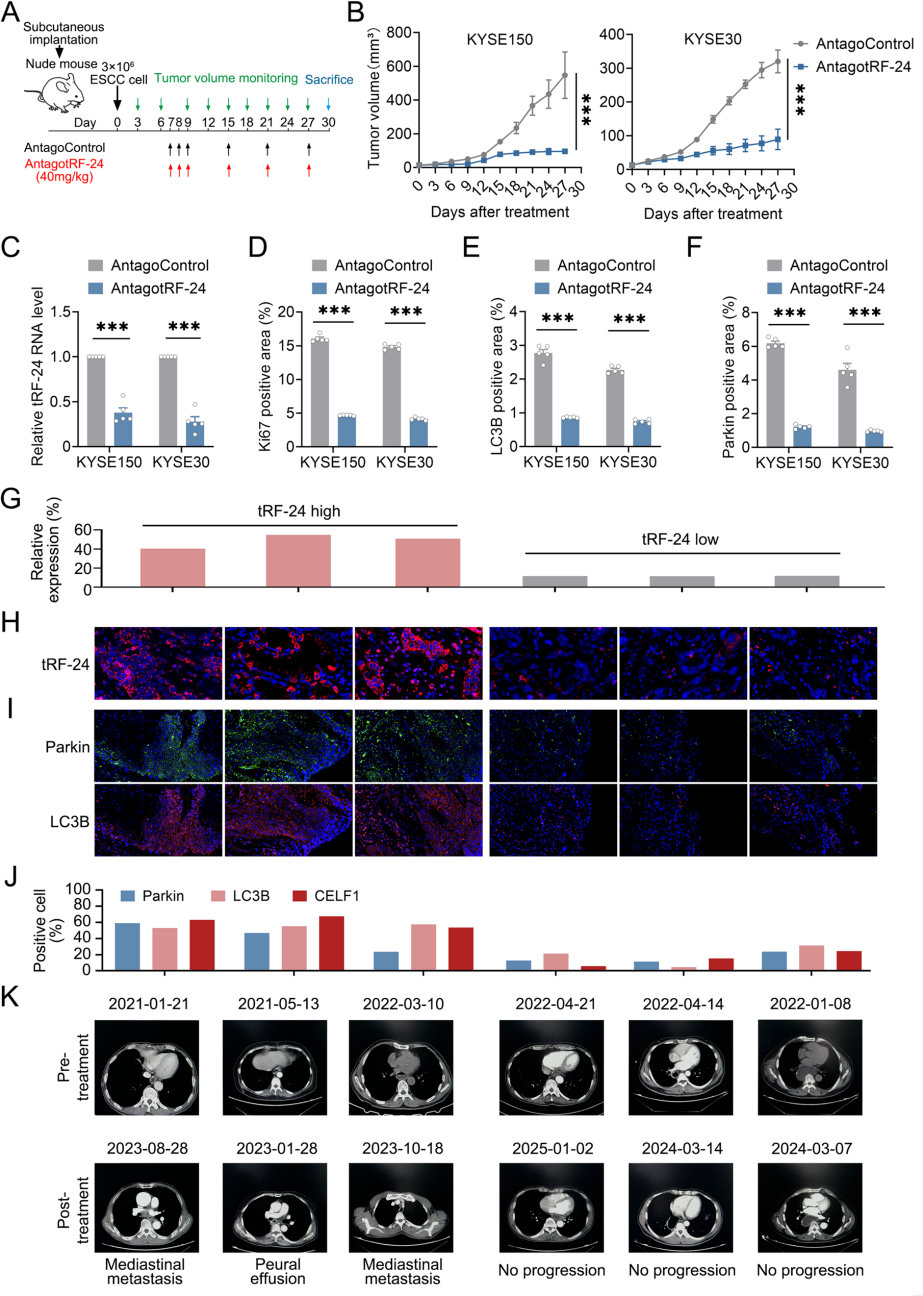
Targeting *tRF-24* shows therapeutic effects in mouse models and its expression correlates with ESCC prognosis (**A**) Schematic of the treatment of mice bearing subcutaneous xenografts derived from ESCC cells with antagotRF-24 or antagoControl by intravenous injection. Colored arrows indicate dosing and tumor radiance monitoring time points. **B** AntagotRF-24 treatment significantly inhibited subcutaneous xenograft growth in mice (*n* = 5). **C** *tRF-24* levels in subcutaneous xenografts from mice with different treatments. **D–F** IHC analysis of Ki-67, LC3B and Parkin levels in ESCC subcutaneous xenografts with different treatments. Positivity was determined by threshold-based segmentation of IHC staining. Data in (**B-F**) represent mean ± SEM of 5 mice per group. *P < 0.05, ***P < 0.001 by Student’s *t* test. **G–H** The percentage (**G**) and images (**H**) of *tRF-24*-positive cells in representative ESCC patients by RNA FISH. Nuclei were counterstained with DAPI. Scale bars: 20 μm. **I**,** J** Immunofluorescence analysis of LC3B and Parkin levels within tumor tissues from these representative patients. Representative mIHC staining images (**I**) and quantification (**J**). Scale bars: 50 μm. **K** Representative follow-up CT images from these representative patients

Given the oncogenic roles of *tRF-24* in ESCC pathogenesis, we developed xenograft models with ESCC cells. We then administered the *tRF-24* antagomir starting on day 7 post-implantation (Fig. 7A). Systemic administration of antagotRF-24 (40 mg/kg, i.v.) significantly suppressed tumor growth compared to control mice (Fig. 7B, Supplementary Fig. 14 A). Administration of antagotRF-24 significantly decreased *tRF-24* levels in the xenograft tumors but had no effect on body weight of the mice (Fig. 7C and Supplementary Fig. 14B), suggesting no apparent toxicity to the animals. Analysis of effectors downstream of *tRF-24* in the xenograft tumors revealed that antagotRF-24 treatment substantially decreased the levels of *BIN1-L* and *BECN1-α* compared to the control group treated with antagoControl (Supplementary Fig. 14 C). Quantitative IHC analysis showed a significant reduction in the proportions of Ki67, LC3B and Parkin in antagotRF-24-treated tumors compared to the antagoControl group (Fig. 7D–F, Supplementary Fig. 14D). Overall, our findings suggest that antagotRF-24 is a potential therapeutic candidate for ESCC, while also demonstrating a favorable safety profile in mouse models.

To assess clinical applicability, we performed fluorescence in situ hybridization (FISH) assays on tumor tissues from six ESCC patients with primary lesions. According to the validated cutoff (≥ 25% positive cells), patients were divided into two cohorts including *tRF-24* high (*n* = 3) and *tRF-24* low (*n* = 3) (Fig. 7G-H). Multiplex immunohistochemistry (mIHC) analysis of six clinical specimens showed that the *tRF-24* high-expressing group had significantly higher levels of LC3 and Parkin than the low-expressing group (Fig. 7I-J). Longitudinal CT imaging showed a longer time interval to progression in the *tRF-24* low group compared to the *tRF-24* high group (Fig. 7K). Overall, these findings indicate that *tRF-24* displays promising preclinical therapeutic efficacy and potential for clinical applications in ESCC patients.

## Discussion

**Fig. 8:**
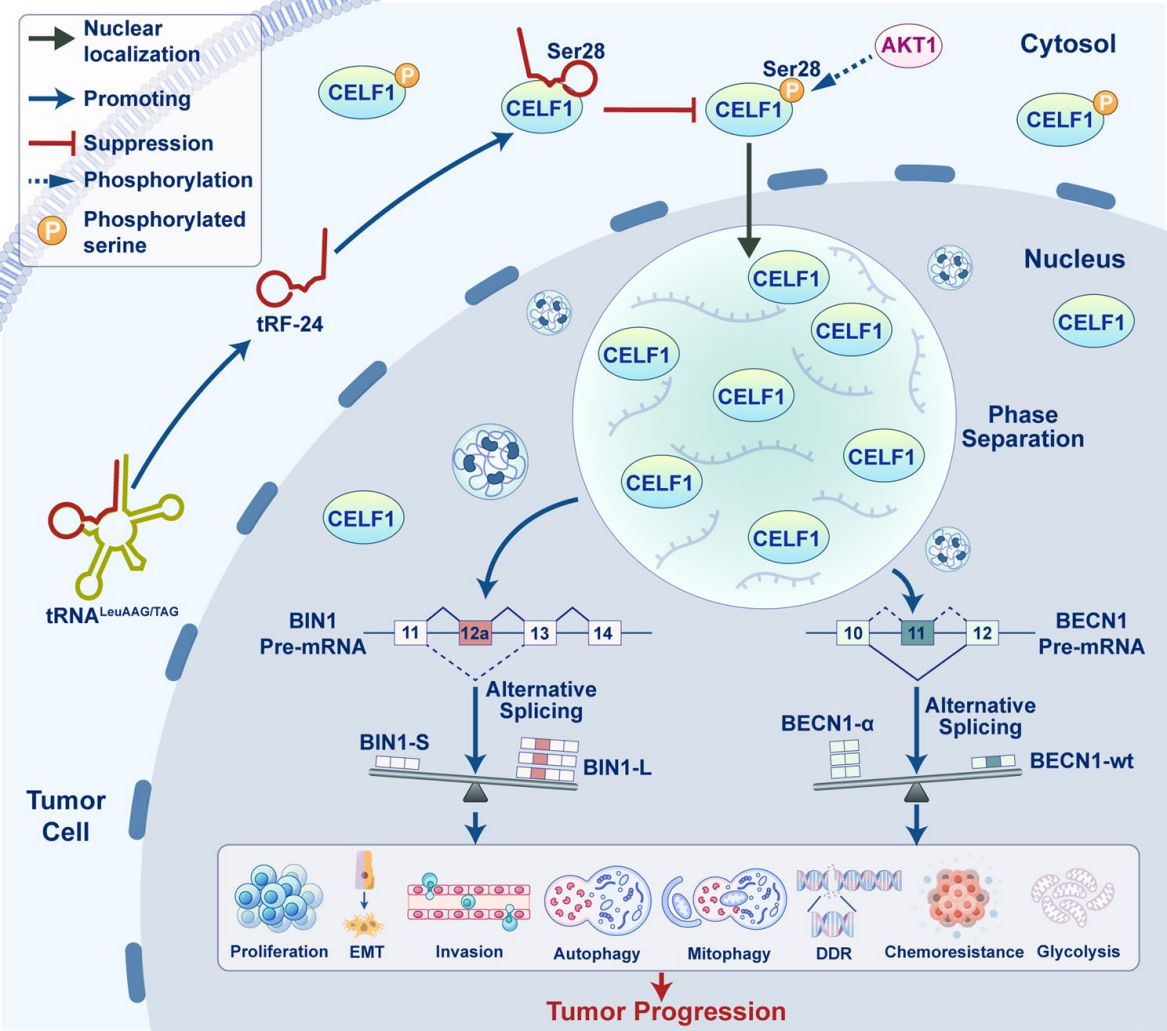
Schematic model.*tRF-24* binds to CELF1 and blocks AKT1-mediated CELF1 phosphorylation at Ser28, enhancing its nuclear translocation. Nuclear CELF1 undergoes phase separation, further enhancing its alternative splicing activity. The resulting overproduction of *BIN1-L* and *BECN1-α* isoforms drives abnormal proliferation, epithelial-mesenchymal transition (EMT), migration, autophagy, mitophagy, DNA damage repair (DDR), chemoresistance and glycolysis in ESCC

In this study, we identified *tRF-24* as a novel oncogenic driver of ESCC. Clinical analysis revealed that the expression of *tRF-24* was higher in ESCC tissues than adjacent normal tissues, and its elevated levels were strongly associated with more advanced clinical stage and poorer overall survival. Functionally, *tRF-24* promoted malignant behavior in ESCC cells by enhancing proliferation and migration, while also activating autophagy, mitophagy, DNA damage repair, cisplatin resistance and glycolytic metabolism. Mechanistically, we found that *tRF-24* directly bound to Ser28 in the RRM1 domain of CELF1, blocking AKT1-mediated phosphorylation at this residue. This inhibition promoted the nuclear translocation of CELF1, leading to its accumulation in the nucleus and triggering its LLPS. The resulting LLPS-induced condensates facilitated CELF1-mediated alternative splicing of *BIN1* and *BECN1* pre-mRNAs, preferentially producing pro-oncogenic *BIN1-L* and pro-autophagic/mitophagic *BECN1-α* isoforms (Fig. 8). In vivo experiments demonstrated the therapeutic potential of the *tRF-24* antagomir, which resulted in significant tumor suppression compared to control. Our findings define the *tRF-24*-CELF1-*BIN1*/*BECN1* regulatory axis in ESCC and highlighted the potential of targeting *tRF-24* in clinical applications.

Autophagy is an evolutionarily conserved self-degradation process with non-selective and substrate-selective pathways. While the role of non-coding RNAs in autophagy has gained increasing attention, emerging studies demonstrate that their cancer-specific regulatory networks are more complex than previously understood. LncRNAs, microRNAs, circRNAs and snoRNAs have emerged as key regulators of autophagy. They modulate autophagy through various mechanisms, shaping context-dependent networks across different tumor types [58–60]. Mitochondrial quality control through selective autophagy (mitophagy) is a pivotal cellular defense mechanism. The PINK1/Parkin pathway coordinates this process by sequentially phosphorylating ubiquitin on damaged mitochondria, triggering selective autophagic clearance [31]. Recent evidence shows that the mitochondrial tRNA fragment *mt-tRF3b-LeuTAA* binds to the 3’ UTR of *SENP1*, acting as a miRNA-like suppressor to reduce SENP1 protein levels. This regulatory mechanism disrupts its downstream effectors and impairs Parkin/PINK1-mediated mitophagy in chondrocytes [61]. However, no relevant reports have been found in cancer. The present study reveals, for the first time to our knowledge, that certain tRFs (i.e., *tRF-24*) can affect the alternative splicing of autophagy and mitophagy-related genes by binding to RBPs. These findings substantially expand our knowledge of novel regulatory mechanisms that extend the paradigms of autophagy and mitophagy.

Our study establishes a novel connection between the oncogenic activity of *tRF-24* in ESCC and LLPS. Current models suggest that longer RNA transcripts (average length about 1.9 kb) primarily drive LLPS through multivalent interactions [62]. In contrast, the 24-nucleotide *tRF-24* unexpectedly regulates LLPS. Mechanistically, *tRF-24* regulates LLPS dynamics by redistributing CELF1 between the nucleus and cytoplasm. This mechanism differs from established models of RNA-mediated phase separation, which typically involve Coulombic complementarity [63] or scaffold formation [54]. These findings add a new dimension to tRNA fragment biology and provide the first evidence of tsRNA involvement in LLPS-mediated cellular pathophysiology. CELF1 is an RNA-binding protein that regulates alternative splicing, mRNA stability, and translation [33]. Numerous studies have established that CELF1 is phosphorylated at specific residues, with these modifications determining its functions. Phosphorylation at Ser28 enhances cytoplasmic localization, while phosphorylation at Ser302 promotes the assembly of the translational complex with eIF2. These findings collectively highlight the diverse regulatory roles of CELF1 post-translational modifications [35]. For the first time, our data demonstrate that *tRF-24* binds to CELF1, inhibiting phosphorylation at the Ser28 site through steric hindrance, which results in the nuclear accumulation of CELF1. Additionally, we present an innovative finding on the role of LLPS in enhancing CELF1-mediated alternative splicing. These novel discoveries expand existing knowledge by introducing a new aspect of tRNA fragment biology.

These findings establish the *tRF-24*-CELF1-*BIN1*/*BECN1* axis as a key regulator of ESCC progression and the therapeutic potential of targeting this specific tRF molecule was confirmed in xenograft models, where treatment with antagotRF-24 reduced subcutaneous tumor volume compared to controls. Furthermore, no significant changes in body weight were observed, indicating favorable safety profile of this customized reagent. Notably, the validation of this pathway in clinical ESCC samples yielded encouraging results. ESCC patients with high *tRF-24* expression, who were randomly selected for follow-up, showed a shorter postoperative recurrence interval. The analysis of clinical samples revealed a strong correlation between the expression of *tRF-24* and key targets in this pathway. All these conclusions provide preclinical evidence for RNA-based therapies in the comprehensive treatment of ESCC.

This study advances our understanding of tRNA-derived fragments (tRFs) and their roles in ESCC. However, several limitations should be considered. First, while emerging evidence suggests that tRNA cleavage is regulated by multiple enzymes under stress conditions, the precise biogenesis mechanism of *tRF-24* remains unclear. Second, our findings directly implicate *tRF-24* in regulating CELF1 nucleocytoplasmic transport. Given the known role of CELF1 in regulating alternative splicing, further studies should explore potential co-factors involved in its LLPS.

## Conclusion

We identify *tRF-24* as a novel oncogenic tRNA-derived fragment that critically regulates ESCC progression. Our mechanistic studies elucidate how *tRF-24* drives tumor growth, metastasis and chemoresistance through CELF1 phase separation-mediated alternative splicing. Notably, targeting *tRF-24* with an antagomir significantly suppressed tumor progression in xenograft models. These findings position *tRF-24* as both a prognostic biomarker and a therapeutic target, with potential clinical applications in ESCC, providing a rationale for developing tRNA fragment-targeted therapies.

## Supplementary Information

Supplementary Material 1.

## Data Availability

All data needed to evaluate the conclusions are provided herein or in the supplemental material. RNA sequencing data generated in this study have been deposited in the Genome Sequence Archive in BIG Data Center (https://bigd.big.ac.cn/), Beijing Institute of Genomics, Chinese Academy of Sciences, under the accession number: HRA011225. The mass spectrometry proteomics data have been deposited in the ProteomeXchange Consortium (http://proteomecentral.proteomexchange.org) under the dataset identifier PXD063163. All other raw data can be obtained from the corresponding author upon reasonable request.

## Abbreviations

tRFs: tRNA-derived fragments
ESCC: Esophageal squamous cell carcinoma
tRF-24: tRF-24-RPM8309M2S
GEO: Gene Expression Omnibus
EMT: Epithelial-Mesenchymal Transition
LLPS: Liquid-liquid phase separation
ECAR: Extracellular Acidification Rate
tsRNAs: tRNA-derived small RNAs
RBP: RNA-binding protein
AGOs: Argonaute proteins
RISC: RNA-induced silencing complex
3’UTR: 3’ untranslated region
IDRs: Intrinsically disordered regions
CELF1: CUGBP Elav-like family member 1
AKT1: RAC-alpha serine/threonine-protein kinase
BIN1: Myc box-dependent-interacting protein 1
BECN1: Beclin-1
ATM: Kinase ataxia telangiectasia mutated
lncRNAs: Long non-coding RNAs
OS: Overall survival
PFS: Progression-free survival
DIG: Digoxigenin
H&E: Hematoxylin and eosin
IHC: Immunohistochemistry
FISH: Fluorescence in Situ Hybridization
mIHC: Multiplex immunohistochemistry
qRT-PCR: quantitative real-time PCR
MS: mass spectrometry
RIP: RNA immunoprecipitation
RNA-seq: RNA sequencing
FRAP: Fluorescence recovery after photobleaching

## References

[1] Bray F, Laversanne M, Sung H, Ferlay J, Siegel RL, Soerjomataram I, et al. Global cancer statistics 2022: GLOBOCAN estimates of incidence and mortality worldwide for 36 cancers in 185 countries. CA Cancer J Clin. 2024;74(3):229–63. 38572751 10.3322/caac.21834

[2] Han B, Zheng R, Zeng H, Wang S, Sun K, Chen R, et al. Cancer incidence and mortality in China, 2022. J Natl Cancer Center. 2024;4(1):47–53. 39036382 10.1016/j.jncc.2024.01.006 PMC11256708

[3] Matz M, Valkov M, Šekerija M, Luttman S, Caldarella A, Coleman MP, et al. Worldwide trends in esophageal cancer survival, by sub-site, morphology, and sex: an analysis of 696,974 adults diagnosed in 60 countries during 2000–2014 (CONCORD-3). Cancer Commun. 2023;43(9):963–80. 10.1002/cac2.12457 PMC10508138 37488785

[4] Muthukumar S, Li CT, Liu RJ, Bellodi C. Roles and regulation of tRNA-derived small RNAs in animals. Nat Rev Mol Cell Biol. 2024;25(5):359–78. 38182846 10.1038/s41580-023-00690-z

[5] Fu M, Gu J, Wang M, Zhang J, Chen Y, Jiang P, et al. Emerging roles of tRNA-derived fragments in cancer. Mol Cancer. 2023;22(1):30. 36782290 10.1186/s12943-023-01739-5 PMC9926655

[6] Pinzaru AM, Tavazoie SF. Transfer RNAs as dynamic and critical regulators of cancer progression. Nat Rev Cancer. 2023;23(11):746–61. 37814109 10.1038/s41568-023-00611-4

[7] Huang B, Yang H, Cheng X, Wang D, Fu S, Shen W, et al. TRf/miR-1280 suppresses stem cell-like cells and metastasis in colorectal cancer. Cancer Res. 2017;77(12):3194–206. 28446464 10.1158/0008-5472.CAN-16-3146

[8] Maute RL, Schneider C, Sumazin P, Holmes A, Califano A, Basso K, et al. tRNA-derived microRNA modulates proliferation and the DNA damage response and is down-regulated in B cell lymphoma. Proc Natl Acad Sci U S A. 2013;110(4):1404–9. 23297232 10.1073/pnas.1206761110 PMC3557069

[9] Chen Q, Yan M, Cao Z, Li X, Zhang Y, Shi J, et al. Sperm TsRNAs contribute to intergenerational inheritance of an acquired metabolic disorder. Science. 2016;351(6271):397–400. 26721680 10.1126/science.aad7977

[10] Goodarzi H, Liu X, Nguyen HC, Zhang S, Fish L, Tavazoie SF. Endogenous tRNA-Derived fragments suppress breast cancer progression via YBX1 displacement. Cell. 2015;161(4):790–802. 25957686 10.1016/j.cell.2015.02.053 PMC4457382

[11] Guzzi N, Cieśla M, Ngoc PCT, Lang S, Arora S, Dimitriou M, et al. Pseudouridylation of tRNA-Derived fragments steers translational control in stem cells. Cell. 2018;173(5):1204–e1626. 29628141 10.1016/j.cell.2018.03.008

[12] Pan L, Huang X, Liu ZX, Ye Y, Li R, Zhang J, et al. Inflammatory cytokine-regulated tRNA-derived fragment tRF-21 suppresses pancreatic ductal adenocarcinoma progression. J Clin Invest. 2021. 10.1172/JCI148130. 10.1172/JCI148130 PMC8592549 34779408

[13] Mehta S, Zhang J. Liquid-liquid phase separation drives cellular function and dysfunction in cancer. Nat Rev Cancer. 2022;22(4):239–52. 35149762 10.1038/s41568-022-00444-7 PMC10036213

[14] Zheng LW, Liu CC, Yu KD. Phase separations in oncogenesis, tumor progressions and metastasis: a glance from hallmarks of cancer. J Hematol Oncol. 2023;16(1):123. 38110976 10.1186/s13045-023-01522-5 PMC10726551

[15] Roden C, Gladfelter AS. RNA contributions to the form and function of biomolecular condensates. Nat Rev Mol Cell Biol. 2021;22(3):183–95. 32632317 10.1038/s41580-020-0264-6 PMC7785677

[16] Liu B, Shen H, He J, Jin B, Tian Y, Li W, et al. Cytoskeleton remodeling mediated by circRNA-YBX1 phase separation suppresses the metastasis of liver cancer. Proc Natl Acad Sci U S A. 2023;120(30):e2220296120. 37459535 10.1073/pnas.2220296120 PMC10372620

[17] Obermannová R, Alsina M, Cervantes A, Leong T, Lordick F, Nilsson M, et al. Oesophageal cancer: ESMO clinical practice guideline for diagnosis, treatment and follow-up. Ann Oncol. 2022;33(10):992–1004. 35914638 10.1016/j.annonc.2022.07.003

[18] Chen C, Ridzon DA, Broomer AJ, Zhou Z, Lee DH, Nguyen JT, et al. Real-time quantification of MicroRNAs by stem-loop RT-PCR. Nucleic Acids Res. 2005;33(20):e179. 16314309 10.1093/nar/gni178 PMC1292995

[19] Scherr M, Venturini L, Battmer K, Schaller-Schoenitz M, Schaefer D, Dallmann I, et al. Lentivirus-mediated antagomir expression for specific inhibition of miRNA function. Nucleic Acids Res. 2007;35(22):e149. 18025036 10.1093/nar/gkm971 PMC2190705

[20] Huang Y, Wan Z, Tang Y, Xu J, Laboret B, Nallamothu S, et al. Pantothenate kinase 2 interacts with PINK1 to regulate mitochondrial quality control via acetyl-CoA metabolism. Nat Commun. 2022;13(1):2412. 35504872 10.1038/s41467-022-30178-x PMC9065001

[21] Pliatsika V, Loher P, Magee R, Telonis AG, Londin E, Shigematsu M, et al. MINTbase v2.0: a comprehensive database for tRNA-derived fragments that includes nuclear and mitochondrial fragments from all the cancer genome atlas projects. Nucleic Acids Res. 2018;46(D1):D152–9. 29186503 10.1093/nar/gkx1075 PMC5753276

[22] Wu Q, Zhang W, Wang Y, Min Q, Zhang H, Dong D, et al. MAGE-C3 promotes cancer metastasis by inducing epithelial-mesenchymal transition and immunosuppression in esophageal squamous cell carcinoma. Cancer Commun. 2021;41(12):1354–72. 10.1002/cac2.12203 PMC8696229 34347390

[23] Dong D, Zhou Z, Zhu M, Hou Z, Chen M, Gong J, et al. STN1 facilitates metastasis by promoting transcription of EMT-activator ZEB1 in pancreatic cancer. Nat Commun. 2025;16(1):7815. 40841373 10.1038/s41467-025-63083-0 PMC12370990

[24] Fan C, Cats D, Selle M, Khorosjutina O, Dhanjal S, Schmierer B, et al. Smad3 and p300 complex scaffolding by long non-coding RNA LIMD1-AS1 promotes TGF-β-induced breast cancer cell plasticity. Nucleic Acids Res. 2025. 10.1093/nar/gkaf841. 10.1093/nar/gkaf841 PMC12400928 40889156

[25] Zhang W, Peng Y, Zhou M, Qian L, Che Y, Chen J, et al. RBM14 enhances transcriptional activity of p23 regulating CXCL1 expression to induce lung cancer metastasis. Acta Pharm Sin B. 2025;15(6):3059–72. 40654360 10.1016/j.apsb.2025.03.048 PMC12254758

[26] Liu J, Wu Y, Meng S, Xu P, Li S, Li Y, et al. Selective autophagy in cancer: mechanisms, therapeutic implications, and future perspectives. Mol Cancer. 2024;23(1):22. 38262996 10.1186/s12943-024-01934-y PMC10807193

[27] La Belle Flynn A, Calhoun BC, Sharma A, Chang JC, Almasan A, Schiemann WP. Autophagy inhibition elicits emergence from metastatic dormancy by inducing and stabilizing Pfkfb3 expression. Nat Commun. 2019;10(1):3668. 31413316 10.1038/s41467-019-11640-9 PMC6694140

[28] Zhan Z, Xie X, Cao H, Zhou X, Zhang XD, Fan H, et al. Autophagy facilitates TLR4- and TLR3-triggered migration and invasion of lung cancer cells through the promotion of TRAF6 ubiquitination. Autophagy. 2014;10(2):257–68. 24321786 10.4161/auto.27162 PMC5396095

[29] Yu Y, Song Y, Cheng L, Chen L, Liu B, Lu D, et al. CircCEMIP promotes anoikis-resistance by enhancing protective autophagy in prostate cancer cells. J Exp Clin Cancer Res. 2022;41(1):188. 35655258 10.1186/s13046-022-02381-7 PMC9161511

[30] Han JH, Kim YK, Kim H, Lee J, Oh MJ, Kim SB, et al. Snail acetylation by autophagy-derived acetyl-coenzyme A promotes invasion and metastasis of *KRAS*-*LKB1* co-mutated lung cancer cells. Cancer Commun. 2022;42(8):716–49. 10.1002/cac2.12332 PMC9395322 35838183

[31] Picca A, Faitg J, Auwerx J, Ferrucci L, D’Amico D. Mitophagy in human health, ageing and disease. Nat Metab. 2023;5(12):2047–61. 38036770 10.1038/s42255-023-00930-8 PMC12159423

[32] Philips AV, Timchenko LT, Cooper TA. Disruption of splicing regulated by a CUG-binding protein in myotonic dystrophy. Science. 1998;280(5364):737–41. 9563950 10.1126/science.280.5364.737

[33] Qin WJ, Shi JJ, Chen RY, Li CY, Liu YJ, Lu JF, et al. Curriculum vitae of CUG binding protein 1 (CELF1) in homeostasis and diseases: a systematic review. Cell Mol Biol Lett. 2024;29(1):32. 38443798 10.1186/s11658-024-00556-y PMC10916161

[34] Hornbeck PV, Zhang B, Murray B, Kornhauser JM, Latham V, Skrzypek E. PhosphoSitePlus, 2014: mutations, PTMs and recalibrations. Nucleic Acids Res. 2015;43:D512–520. 25514926 10.1093/nar/gku1267 PMC4383998

[35] Huichalaf C, Sakai K, Jin B, Jones K, Wang GL, Schoser B, et al. Expansion of CUG RNA repeats causes stress and inhibition of translation in myotonic dystrophy 1 (DM1) cells. FASEB J. 2010;24(10):3706–19. 20479119 10.1096/fj.09-151159 PMC2996918

[36] Salisbury E, Sakai K, Schoser B, Huichalaf C, Schneider-Gold C, Nguyen H, et al. Ectopic expression of Cyclin D3 corrects differentiation of DM1 myoblasts through activation of RNA CUG-binding protein, CUGBP1. Exp Cell Res. 2008;314(11–12):2266–78. 18570922 10.1016/j.yexcr.2008.04.018 PMC2494712

[37] Li Y, Liu Y, Yu XY, Xu Y, Pan X, Sun Y, et al. Membraneless organelles in health and disease: exploring the molecular basis, physiological roles and pathological implications. Signal Transduct Target Ther. 2024;9(1):305. 39551864 10.1038/s41392-024-02013-w PMC11570651

[38] Riback JA, Zhu L, Ferrolino MC, Tolbert M, Mitrea DM, Sanders DW, et al. Composition-dependent thermodynamics of intracellular phase separation. Nature. 2020;581(7807):209–14. 32405004 10.1038/s41586-020-2256-2 PMC7733533

[39] Li RH, Tian T, Ge QW, He XY, Shi CY, Li JH, et al. A phosphatidic acid-binding lncRNA SNHG9 facilitates LATS1 liquid-liquid phase separation to promote oncogenic YAP signaling. Cell Res. 2021;31(10):1088–105. 34267352 10.1038/s41422-021-00530-9 PMC8486796

[40] Dujardin G, Buratti E, Charlet-Berguerand N, Martins de Araujo M, Mbopda A, Le Jossic-Corcos C, et al. CELF proteins regulate CFTR pre-mRNA splicing: essential role of the divergent domain of ETR-3. Nucleic Acids Res. 2010;38(20):7273–85. 20631008 10.1093/nar/gkq573 PMC2978352

[41] Chang MY, Boulden J, Katz JB, Wang L, Meyer TJ, Soler AP, et al. Bin1 ablation increases susceptibility to cancer during aging, particularly lung cancer. Cancer Res. 2007;67(16):7605–12. 17699764 10.1158/0008-5472.CAN-07-1100

[42] Chang MY, Boulden J, Sutanto-Ward E, Duhadaway JB, Soler AP, Muller AJ, et al. Bin1 ablation in mammary gland delays tissue remodeling and drives cancer progression. Cancer Res. 2007;67(1):100–7. 17210688 10.1158/0008-5472.CAN-06-2742

[43] Ganesan S, MYC. PARP1, and chemoresistance: BIN there, done that? Sci Signal. 2011;4(166):pe15. 21447796 10.1126/scisignal.2001946

[44] Hu Z, Dong L, Li S, Li Z, Qiao Y, Li Y, et al. Splicing regulator p54(nrb) /non-POU domain-containing octamer-binding protein enhances carcinogenesis through oncogenic isoform switch of MYC box-dependent interacting protein 1 in hepatocellular carcinoma. Hepatology. 2020;72(2):548–68. 31815296 10.1002/hep.31062

[45] Wang Z, Xiong S, Wu Z, Wang X, Gong Y, Zhu WG, et al. VCP/p97 ufmylation stabilizes BECN1 and facilitates the initiation of autophagy. Autophagy. 2024;20(9):2041–54. 38762759 10.1080/15548627.2024.2356488 PMC11346537

[46] Gelmetti V, De Rosa P, Torosantucci L, Marini ES, Romagnoli A, Di Rienzo M, et al. PINK1 and BECN1 relocalize at mitochondria-associated membranes during mitophagy and promote ER-mitochondria tethering and autophagosome formation. Autophagy. 2017;13(4):654–69. 28368777 10.1080/15548627.2016.1277309 PMC5388214

[47] Maheshwari C, Vidoni C, Titone R, Castiglioni A, Lora C, Follo C, et al. Isolation, characterization, and autophagy function of BECN1-splicing isoforms in cancer cells. Biomolecules. 2022. 10.3390/biom12081069. 10.3390/biom12081069 PMC9405542 36008963

[48] Folk WP, Kumari A, Iwasaki T, Pyndiah S, Johnson JC, Cassimere EK, et al. Loss of the tumor suppressor BIN1 enables ATM Ser/Thr kinase activation by the nuclear protein E2F1 and renders cancer cells resistant to cisplatin. J Biol Chem. 2019;294(14):5700–19. 30733337 10.1074/jbc.RA118.005699 PMC6462522

[49] Lin J, Wang X, Zhai S, Shi M, Peng C, Deng X, et al. Hypoxia-induced exosomal circPDK1 promotes pancreatic cancer glycolysis via c-myc activation by modulating miR-628-3p/BPTF axis and degrading BIN1. J Hematol Oncol. 2022;15(1):128. 36068586 10.1186/s13045-022-01348-7 PMC9450374

[50] Huo Y, Cai H, Teplova I, Bowman-Colin C, Chen G, Price S, et al. Autophagy opposes p53-mediated tumor barrier to facilitate tumorigenesis in a model of PALB2-associated hereditary breast cancer. Cancer Discov. 2013;3(8):894–907. 23650262 10.1158/2159-8290.CD-13-0011 PMC3740014

[51] Zheng K, Li Y, Wang S, Wang X, Liao C, Hu X, et al. Inhibition of autophagosome-lysosome fusion by ginsenoside Ro via the ESR2-NCF1-ROS pathway sensitizes esophageal cancer cells to 5-fluorouracil-induced cell death via the CHEK1-mediated DNA damage checkpoint. Autophagy. 2016;12(9):1593–613. 27310928 10.1080/15548627.2016.1192751 PMC5082787

[52] Niu X, You Q, Hou K, Tian Y, Wei P, Zhu Y, et al. Autophagy in cancer development, immune evasion, and drug resistance. Drug Resist Updat. 2025;78:101170. 39603146 10.1016/j.drup.2024.101170

[53] Sakamuro D, Elliott KJ, Wechsler-Reya R, Prendergast GC. BIN1 is a novel MYC-interacting protein with features of a tumour suppressor. Nat Genet. 1996;14(1):69–77. 8782822 10.1038/ng0996-69

[54] Wang J, Fan Y, Luo G, Xiong L, Wang L, Wu Z, et al. Nuclear condensates of WW domain-containing adaptor with coiled-coil regulate mitophagy via alternative splicing. Adv Sci. 2025. 10.1002/advs.202406759. 10.1002/advs.202406759 PMC11904943 39840526

[55] Kim GH, Kwon I. Distinct roles of hnRNPH1 low-complexity domains in splicing and transcription. Proc Natl Acad Sci U S A. 2021;118:50. 10.1073/pnas.2109668118 PMC8685725 34873036

[56] Xin D, Gai X, Ma Y, Li Z, Li Q, Yu X. Pre-rRNA facilitates TopBP1-mediated DNA double-strand break response. Adv Sci. 2023;10(28):e2206931. 10.1002/advs.202206931 PMC10558638 37582658

[57] Wei Y, Luo H, Yee PP, Zhang L, Liu Z, Zheng H, et al. Paraspeckle protein NONO promotes TAZ phase separation in the nucleus to drive the oncogenic transcriptional program. Adv Sci. 2021;8(24):e2102653. 10.1002/advs.202102653 PMC8693076 34716691

[58] Ghafouri-Fard S, Shoorei H, Mohaqiq M, Majidpoor J, Moosavi MA, Taheri M. Exploring the role of non-coding RNAs in autophagy. Autophagy. 2022;18(5):949–70. 33525971 10.1080/15548627.2021.1883881 PMC9196749

[59] Wang Y, Mo Y, Peng M, Zhang S, Gong Z, Yan Q, et al. The influence of circular RNAs on autophagy and disease progression. Autophagy. 2022;18(2):240–53. 33904341 10.1080/15548627.2021.1917131 PMC8942425

[60] Tan Y, Li Y, Tang F. Oncogenic SeRNA functional activation: a novel mechanism of tumorigenesis. Mol Cancer. 2020;19(1):74. 32278350 10.1186/s12943-020-01195-5 PMC7149907

[61] Long D, Deng Z, Zhao X, Xu Y, Li W, Mo X, et al. M(7)G-modified mt-tRF3b-LeuTAA regulates mitophagy and metabolic reprogramming via sumoylation of SIRT3 in chondrocytes. Biomaterials. 2025;314:122903. 39454503 10.1016/j.biomaterials.2024.122903

[62] Khong A, Matheny T, Jain S, Mitchell SF, Wheeler JR, Parker R. The stress granule transcriptome reveals principles of mRNA accumulation in stress granules. Mol Cell. 2017;68(4):808–e205. 29129640 10.1016/j.molcel.2017.10.015 PMC5728175

[63] Niu J, Qiu C, Abbott NL, Gellman SH. Formation of versus recruitment to RNA-rich condensates: controlling effects exerted by peptide side chain identity. J Am Chem Soc. 2022;144(23):10386–95. 35639776 10.1021/jacs.2c02222 PMC9746169

