# Supplementary material for: tRNA-derived fragment tRF-24 drives CELF1 phase separation to promote oncogenic splicing in esophageal squamous cell carcinoma
Source: J Exp Clin Cancer Res. 2025 Nov 17;44:307. doi: 10.1186/s13046-025-03553-x (PMC12621390; doi:10.1186/s13046-025-03553-x)
Supplement: Supplementary file 1 — Supplementary Material 1. [file 13046_2025_3553_MOESM1_ESM.pdf]

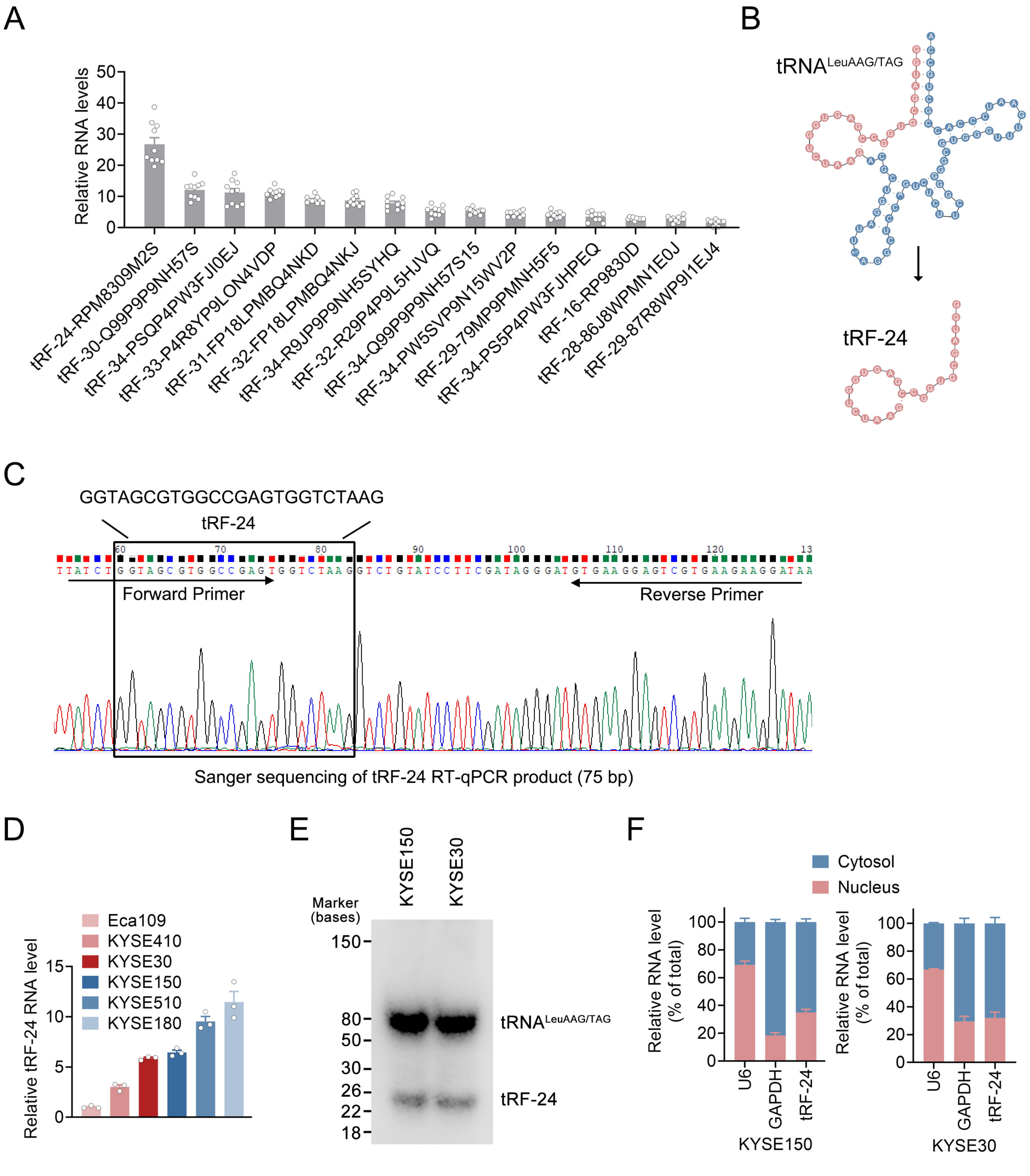
A
Relative RNA levels
50
40
30
20
10
0
tRF-24-RPM8309M2S
tRF-30-Q99P9P9NH57S
tRF-34-PSQP4PW3FJI0EJ
tRF-33-P4R8YP9LON4VDP
tRF-31-FP18LPMBQ4NKD
tRF-32-FP18LPMBQ4NKJ
tRF-34-R9JP9P9NH5SYHQ
tRF-32-R29P4P9L5HJVQ
tRF-34-Q99P9P9NH57S15
tRF-34-PW5SVP9N15WV2P
tRF-29-79MP9PMNH5F5
tRF-34-PS5P4PW3FJHPEQ
tRF-16-RP9830D
tRF-28-86J8WPMN1E0J
tRF-29-87R8WP91EJ4
B
tRNA LeuAAG/TAG
tRF-24
C
GGTAGCGTGGCCGAGTGGTCTAAG
tRF-24
Forward Primer
Reverse Primer
Sanger sequencing of tRF-24 RT-qPCR product (75 bp)
D
Eca109
KYSE410
KYSE30
KYSE150
KYSE510
KYSE180
Relative tRF-24 RNA level
15
10
5
0
E
KYSE150
KYSE30
Marker (bases)
150
80
50
30
26
22
18
tRNA LeuAAG/TAG
tRF-24
F
Cytosol
Nucleus
Relative RNA level (% of total)
100
80
60
40
20
0
U6
GAPDH
tRF-24
KYSE150
KYSE30

Figure S1

Supplementary Figure 1. Identification and characterization of *tRF-24* in ESCC. (**A**) Top 15 tsRNAs showing the most significant increase in expression in 10 ESCC tumors compared to their paired adjacent normal tissues, as assessed by RT-qPCR. (**B**) Diagram and sequence of *tRF-24* derived from mature $tRNA^{LeuAAG/TAG}$. (**C**) Validation of stem-loop RT-qPCR specificity using Sanger sequencing. The sequence within the black box represents tRF-24 and black arrows indicate primers. (**D**) Relative expression of *tRF-24* in six ESCC cell lines measured by RT-qPCR. Data are mean ± SEM from 3 independent experiments. (**E**) Northern blot analysis of *tRF-24* and mature $tRNA^{LeuAAG/TAG}$ abundance in ESCC cells. (**F**) Analysis of subcellular localization by RT-qPCR shows that *tRF-24* is mainly located in the cytoplasm of ESCC cells. *U6* and *GAPDH* serve as nuclear and cytoplasmic markers, respectively. Data are mean ± SEM from 3 independent experiments.

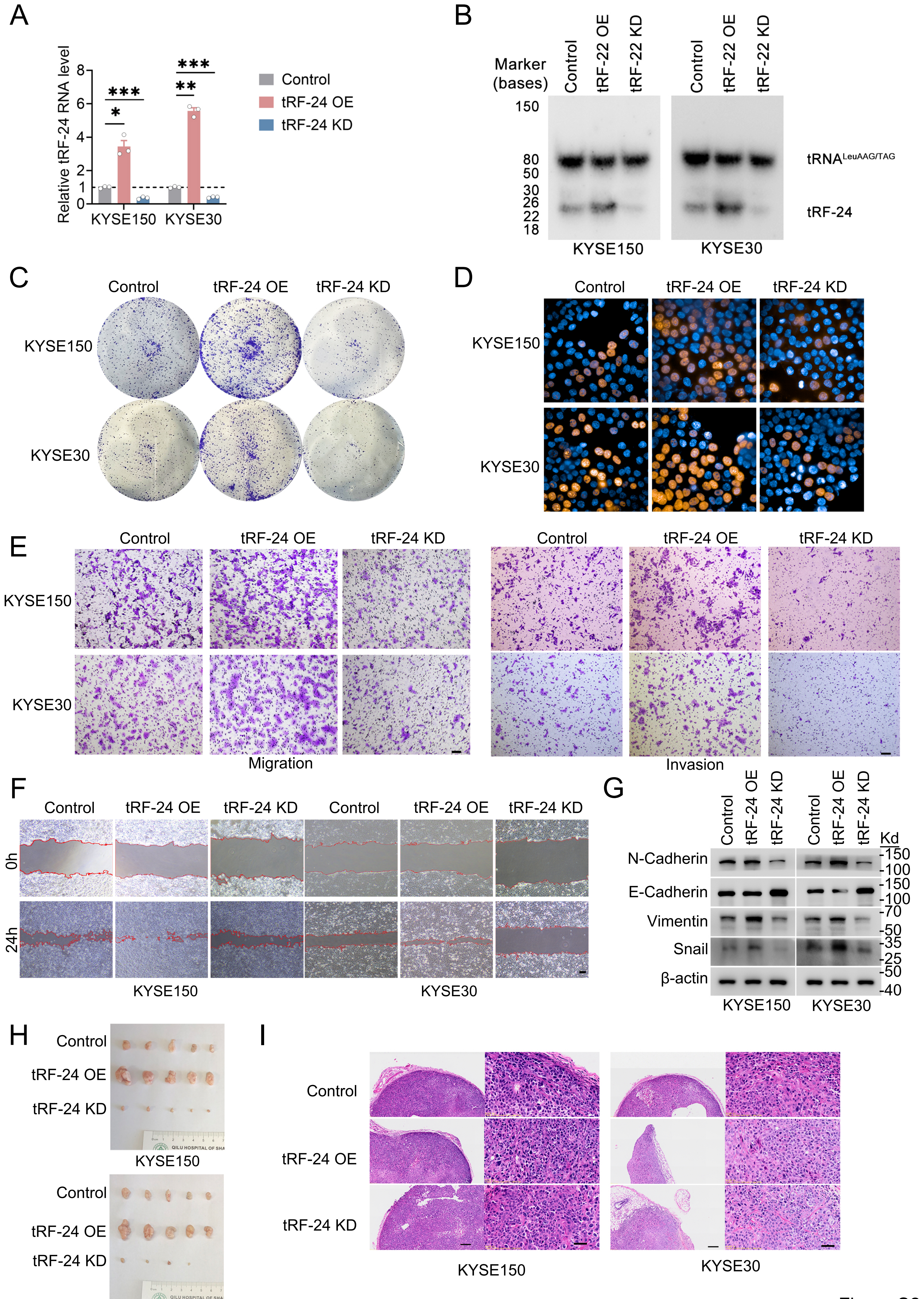
A
Relative tRF-24 RNA level
Control
tRF-24 OE
tRF-24 KD
KYSE150
KYSE30
B
Marker (bases)
Control
tRF-22 OE
tRF-22 KD
150
80
50
30
26
22
18
tRNA LeuAAG/TAG
tRF-24
KYSE150
KYSE30
C
Control
tRF-24 OE
tRF-24 KD
KYSE150
KYSE30
D
Control
tRF-24 OE
tRF-24 KD
KYSE150
KYSE30
E
Control
tRF-24 OE
tRF-24 KD
KYSE150
KYSE30
Migration
Invasion
F
Control
tRF-24 OE
tRF-24 KD
0h
24h
KYSE150
KYSE30
G
Control
tRF-24 OE
tRF-24 KD
Kd
N-Cadherin
E-Cadherin
Vimentin
Snail
β-actin
150
100
70
50
35
25
40
KYSE150
KYSE30
H
Control
tRF-24 OE
tRF-24 KD
KYSE150
KYSE30
I
Control
tRF-24 OE
tRF-24 KD
KYSE150
KYSE30

Figure S2

Supplementary Figure 2. Effects of *tRF*-24 on malignant phenotypes in ESCC cells.

(**A-B**) Relative *tRF-24* levels measured by RT-qPCR (**A**) and northern blot (**B**) in cells with *tRF-24* overexpression (OE) or knockdown (KD). Data are presented as mean ± SEM. **P* < 0.05, ***P* < 0.01, ****P* < 0.001 by one-way ANOVA with Dunnett's T3 multiple comparison test. (**C-F**) Representative images showing the effects of *tRF-24* expression changes on colony formation (**C**), proliferation (**D**), migration and invasion (**E**) and wound healing (**F**) of ESCC cells. Scale bars: 100 µm. (G) Western blotting of EMT-related proteins in in cells with *tRF-24* overexpression (OE) or knockdown (KD). (H) Effects of *tRF-24* expression changes on ESCC xenograft growth in mice (n=5). Shown are images of the subcutaneous xenografts at the end of the experiments. (**I**) Representative histopathological images of xenograft tumors stained with H&E. Scale bars: 500 and 100 µm, respectively.

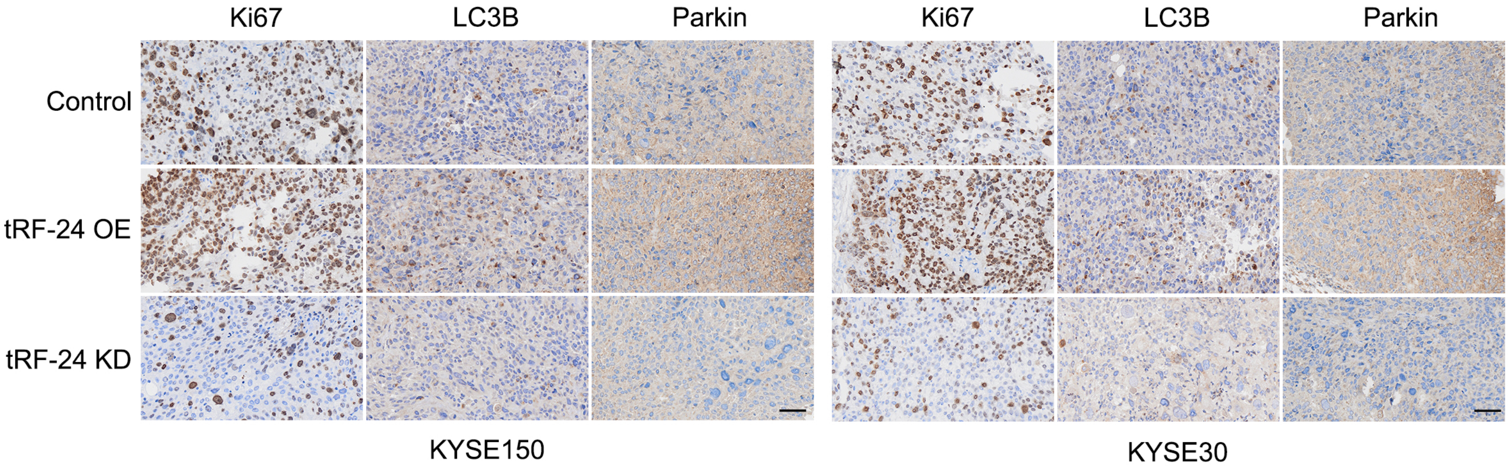
Ki67
LC3B
Parkin
Ki67
LC3B
Parkin
Control
tRF-24 OE
tRF-24 KD
KYSE150
KYSE30

Figure S3

Supplementary Figure 3. ***tRF-24*** **promotes autophagy and mitophagy in ESCC.**

Representative immunohistochemical images showing Ki67, LC3B and Parkin expression in ESCC xenografts. Scale bars: 50 μm. Refer to **Figure 2F**.

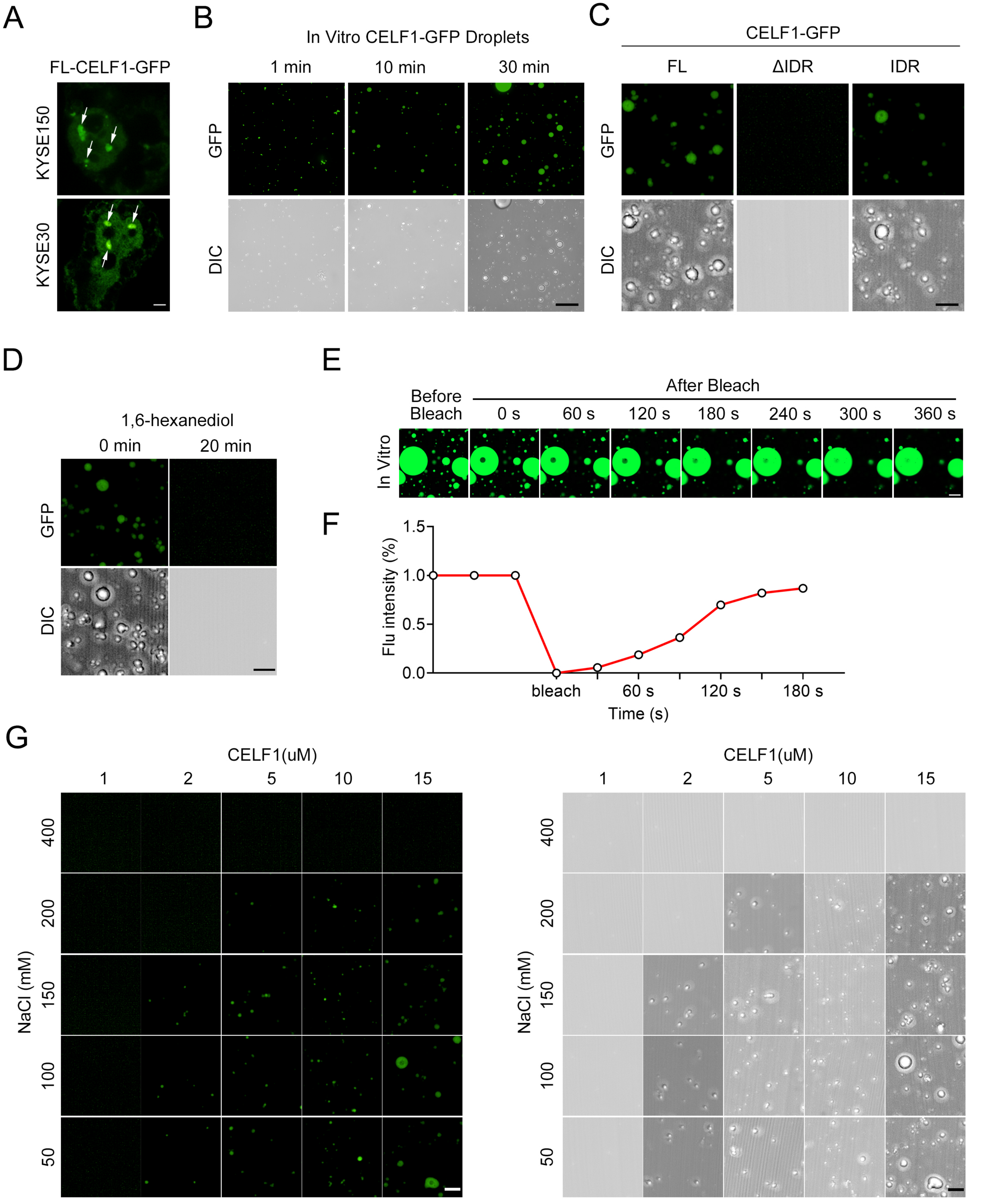
A
FL-CELF1-GFP
KYSE150
KYSE30
B
In Vitro CELF1-GFP Droplets
1 min
10 min
30 min
GFP
DIC
C
CELF1-GFP
FL
ΔIDR
IDR
GFP
DIC
D
1,6-hexanediol
0 min
20 min
GFP
DIC
E
Before Bleach
After Bleach
0 s
60 s
120 s
180 s
240 s
300 s
360 s
In Vitro
F
Flu intensity (%)
1.5
1.0
0.5
0.0
bleach
60 s
120 s
180 s
Time (s)
G
CELF1(uM)
1
2
5
10
15
NaCl (mM)
400
200
150
100
50

Figure S4

Supplementary Figure 4. CELF1 undergoes LLPS *in vitro.*

(**A**) CELF1-GFP forms nuclear puncta in ESCC cells. (**B**) Small droplets fuse into larger ones over time *in vitro*. Scale bars: 5 μm. (**C**) Phase separation assay of truncation mutants of CELF1 *in vitro*. FL-CELF1-GFP phase separated into liquid-like droplets, whereas △IDR-CELF1-GFP did not. Scale bars: 5 μm. (**D**) Effects of 5 % 1,6-hexanediol on CELF1 droplets *in vitro*. (**E-F**) Representative micrographs of CELF1-GFP puncta before and after photobleaching *in vitro* (**E**). Quantification of fluorescence intensity recovery in the bleached region of CELF1 puncta (**F**). (**G**) Representative images of phase separation behavior of different concentrations of purified CELF1-GFP in different concentrations of NaCl (with 5 % PEG8000). Fluorescence (left) and bright-field (right). Scale bars: 5 μm.

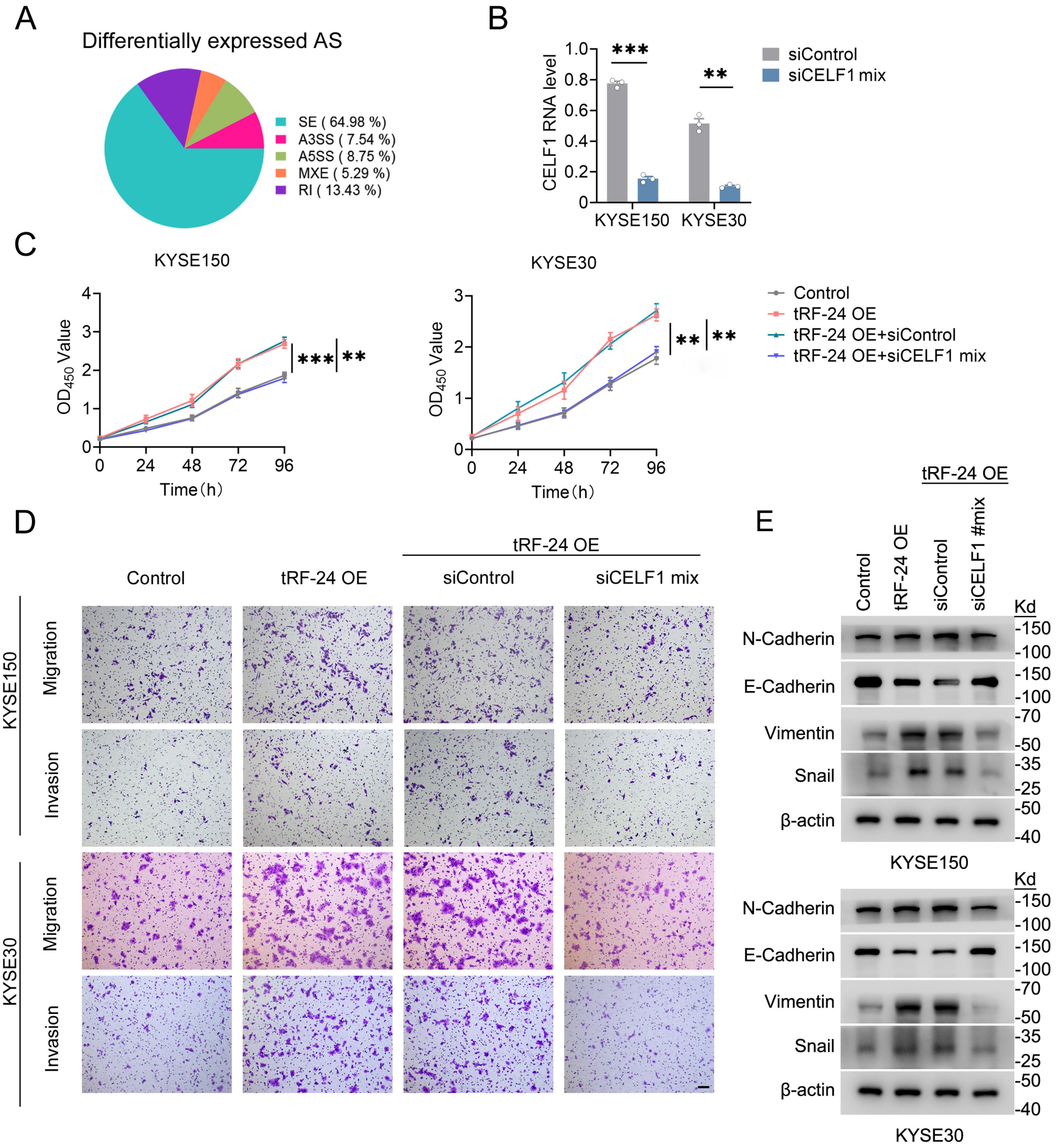
A
Differentially expressed AS
SE (64.98 %)
A3SS (7.54 %)
A5SS (8.75 %)
MXE (5.29 %)
RI (13.43 %)
B
CELF1 RNA level
siControl
siCELF1 mix
KYSE150
KYSE30
C
KYSE150
KYSE30
OD450 Value
Time (h)
Control
tRF-24 OE
tRF-24 OE+siControl
tRF-24 OE+siCELF1 mix
D
tRF-24 OE
Control
tRF-24 OE
siControl
siCELF1 mix
KYSE150
KYSE30
Migration
Invasion
E
tRF-24 OE
Control
tRF-24 OE
siControl
siCELF1 #mix
Kd
N-Cadherin
E-Cadherin
Vimentin
Snail
β-actin
KYSE150
KYSE30

Figure S5

Supplementary Figure 5. CELF1 knockdown rescues oncogenic phenotypes induced by *tRF-24* in ESCC.

(**A**) Endogenous splicing events regulated by *tRF-24* and CELF1. RNA-seq analysis of KYSE150 cells with *tRF-24* or CELF1 overexpression (OE) or control RNA. (**B**) Validation of *CELF1* mRNA levels in cells transfected with *CELF1* siRNA. Data are mean ± SEM from 3 independent experiments. (**C-D**) Effects of CELF1 silencing on the enhanced cell proliferation (**C**), migration and invasion (**D**), EMT (**E**) induced by *tRF-24* overexpression. Scale bars: 100 μm. Data are presented as mean ± SEM from 3 independent experiments. $^{**}P < 0.01$, $^{***}P < 0.001$ by one-way ANOVA with Dunnett's T3 multiple comparison test.

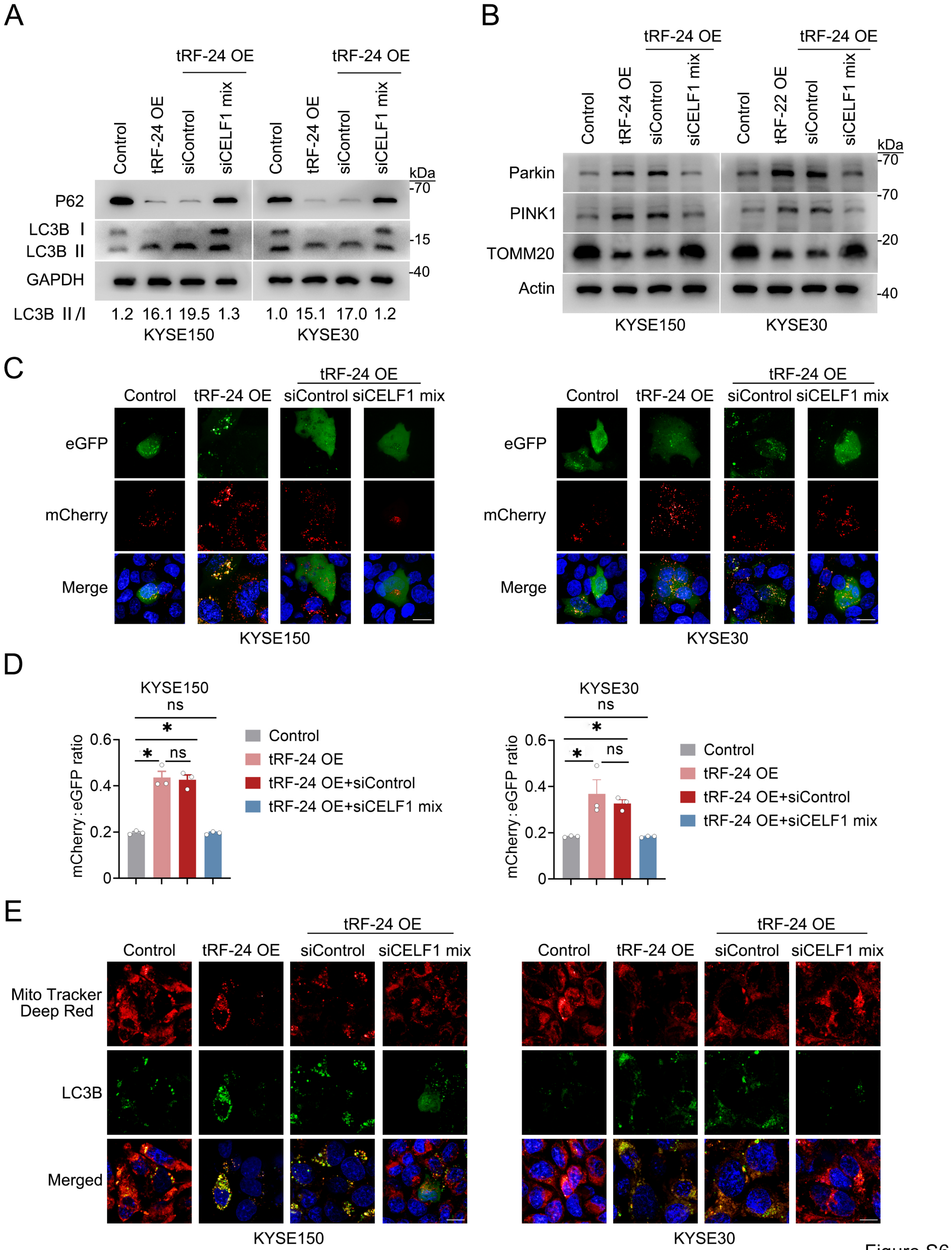
A
tRF-24 OE
tRF-24 OE
Control
tRF-24 OE
siControl
siCELF1 mix
Control
tRF-24 OE
siControl
siCELF1 mix
kDa
-70
P62
LC3B Ⅰ
LC3B Ⅱ
-15
GAPDH
-40
LC3B Ⅱ/Ⅰ
1.2 16.1 19.5 1.3
1.0 15.1 17.0 1.2
KYSE150
KYSE30
B
tRF-24 OE
tRF-24 OE
Control
tRF-24 OE
siControl
siCELF1 mix
Control
tRF-22 OE
siControl
siCELF1 mix
kDa
-70
Parkin
-70
PINK1
-20
TOMM20
Actin
-40
KYSE150
KYSE30
C
Control
tRF-24 OE
tRF-24 OE
siControl siCELF1 mix
eGFP
mCherry
Merge
KYSE150
KYSE30
D
KYSE150
ns
*
*
ns
mCherry:eGFP ratio
0.6
0.4
0.2
0
Control
tRF-24 OE
tRF-24 OE+siControl
tRF-24 OE+siCELF1 mix
KYSE30
E
Control
tRF-24 OE
tRF-24 OE
siControl
siCELF1 mix
Mito Tracker Deep Red
LC3B
Merged
KYSE150
KYSE30

Figure S6

Supplementary Figure 6. CELF1 knockdown rescues *tRF-24*-induced autophagy and mitophagy in ESCC cells.

(**A-B**) Western blot analysis of the effects of CELF1 knockdown on the expression of autophagy-related proteins (**A**) and mitophagy-related proteins (**B**) in *tRF-24* overexpressed KYSE150 cells. (**C-D**) Representative micrographs of autophagic flux following CELF1 silencing in ESCC cells with *tRF-24* overexpression (**C**). Scale bars: 20 μm. Quantitative analysis of the mCherry/GFP fluorescence ratio, which reflects autophagic flux (**D**). Data are presented as mean ± SEM from 3 independent experiments. ns, no significance; $^{*}P < 0.05$ by one-way ANOVA with Dunnett's T3 multiple comparison test. (**E**) Representative micrographs showing mitophagy in ESCC cells with *tRF-24* overexpression following CELF1 knockdown.

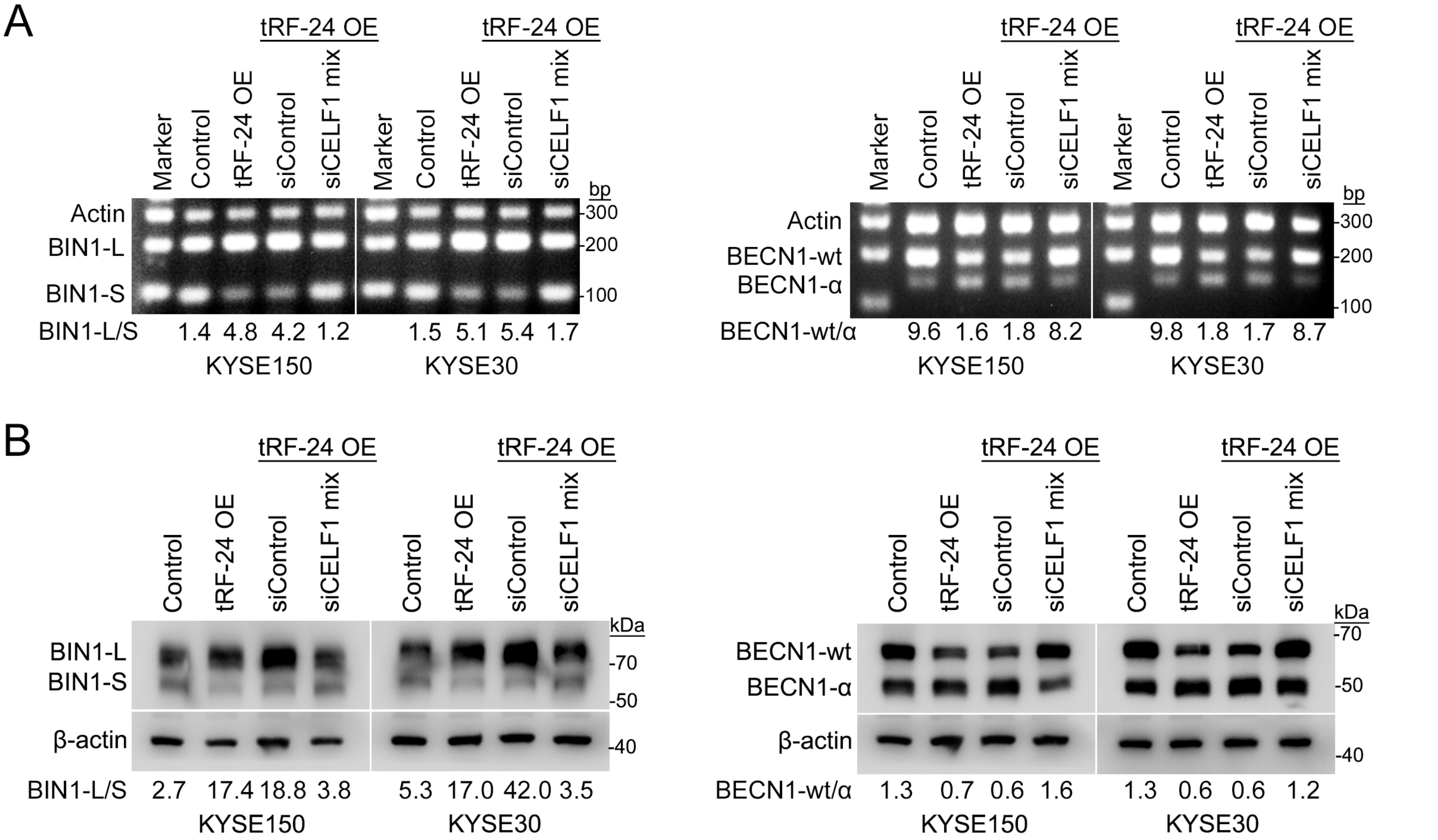
A
tRF-24 OE
Marker
Control
tRF-24 OE
siControl
siCELF1 mix
bp
Actin
BIN1-L
BIN1-S
300
200
100
BIN1-L/S
1.4 4.8 4.2 1.2
1.5 5.1 5.4 1.7
KYSE150
KYSE30
BECN1-wt
BECN1-α
BECN1-wt/α
9.6 1.6 1.8 8.2
9.8 1.8 1.7 8.7
B
kDa
70
50
40
β-actin
2.7 17.4 18.8 3.8
5.3 17.0 42.0 3.5
1.3 0.7 0.6 1.6
1.3 0.6 0.6 1.2

Figure S7

Supplementary Figure 7. CELF1 knockdown rescues *tRF-24*-induced aberrant splicing of *BIN1* and *BECN1* in ESCC.

(**A-B**) Effects of CELF1 knockdown on the ratios of *BIN1* (*BIN1-L/S*) and *BECN1* (*BECN1-wt/α*) mRNAs (**A**) and their protein expression levels (**B**) in cells with *tRF- 24* overexpression.

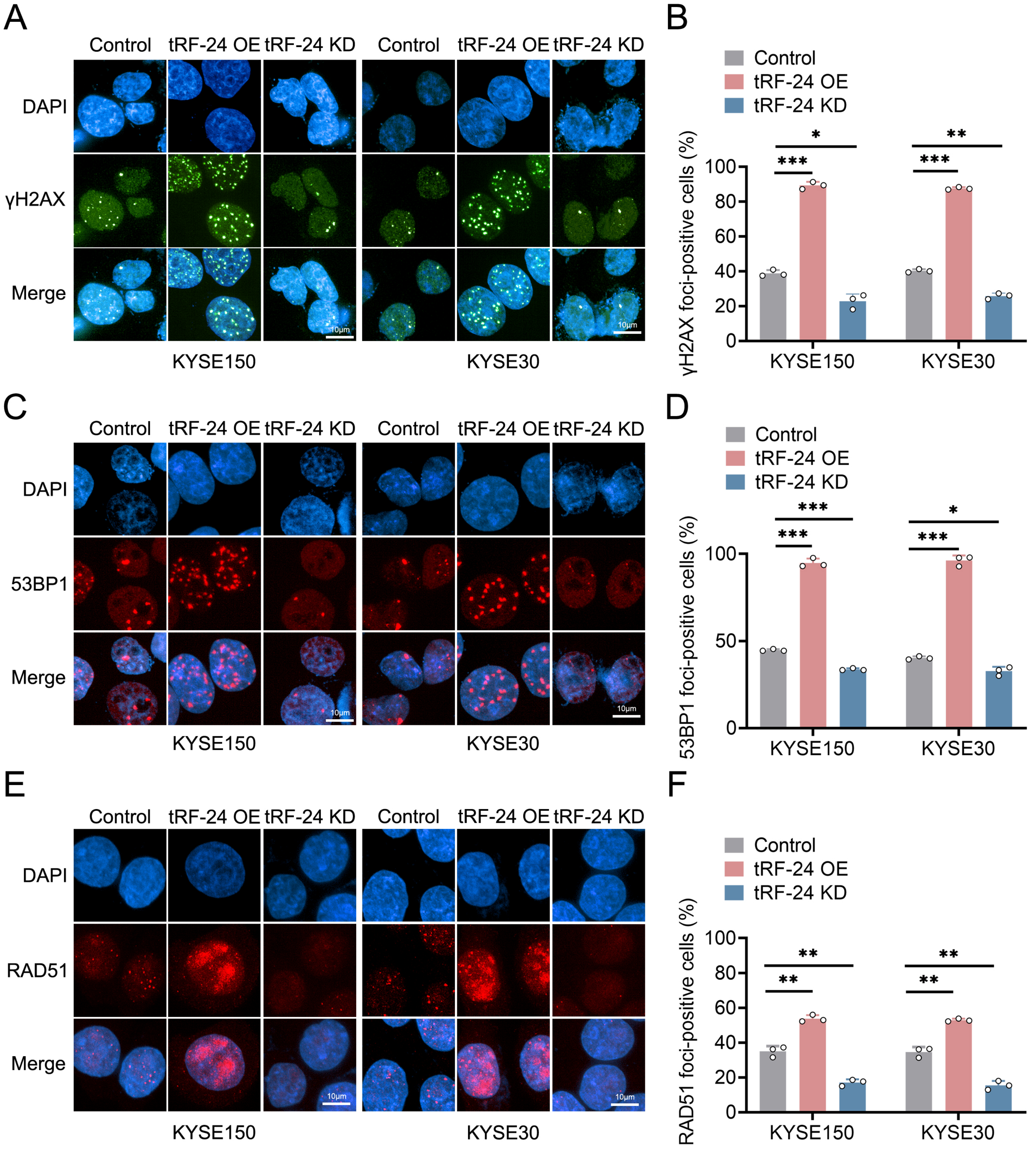

A
Control
tRF-24 OE
tRF-24 KD
DAPI
γH2AX
Merge
10μm
KYSE150
KYSE30
B
Control
tRF-24 OE
tRF-24 KD
γH2AX foci-positive cells (%)
***
*
**
0
20
40
60
80
100
C
53BP1
D
53BP1 foci-positive cells (%)
0
50
100
E
RAD51
F
RAD51 foci-positive cells (%)

Figure S8

Supplementary Figure 8. Effects of *tRF-24* on DSB repair in ESCC cells.

(**A-F**) Representative IF images showing the expression of γH2AX (**A-B**), 53BP1 (**C-D**) and RAD51 (**E-F**) in KYSE150 and KYSE30 cells after 24 h of treatment with 4 μM cisplatin. The percentages of cells with positive nuclei (defined as ≥10 foci per nucleus for γH2AX and 53BP1; ≥5 foci per nucleus for RAD51) are quantified for each cell line and shown. Scale bar, 10 μm. Data are presented as mean ± SEM from 3 independent experiments. *$P < 0.05$, **$P < 0.01$, ***$P < 0.001$ by one-way ANOVA with Dunnett's T3 multiple comparison test.

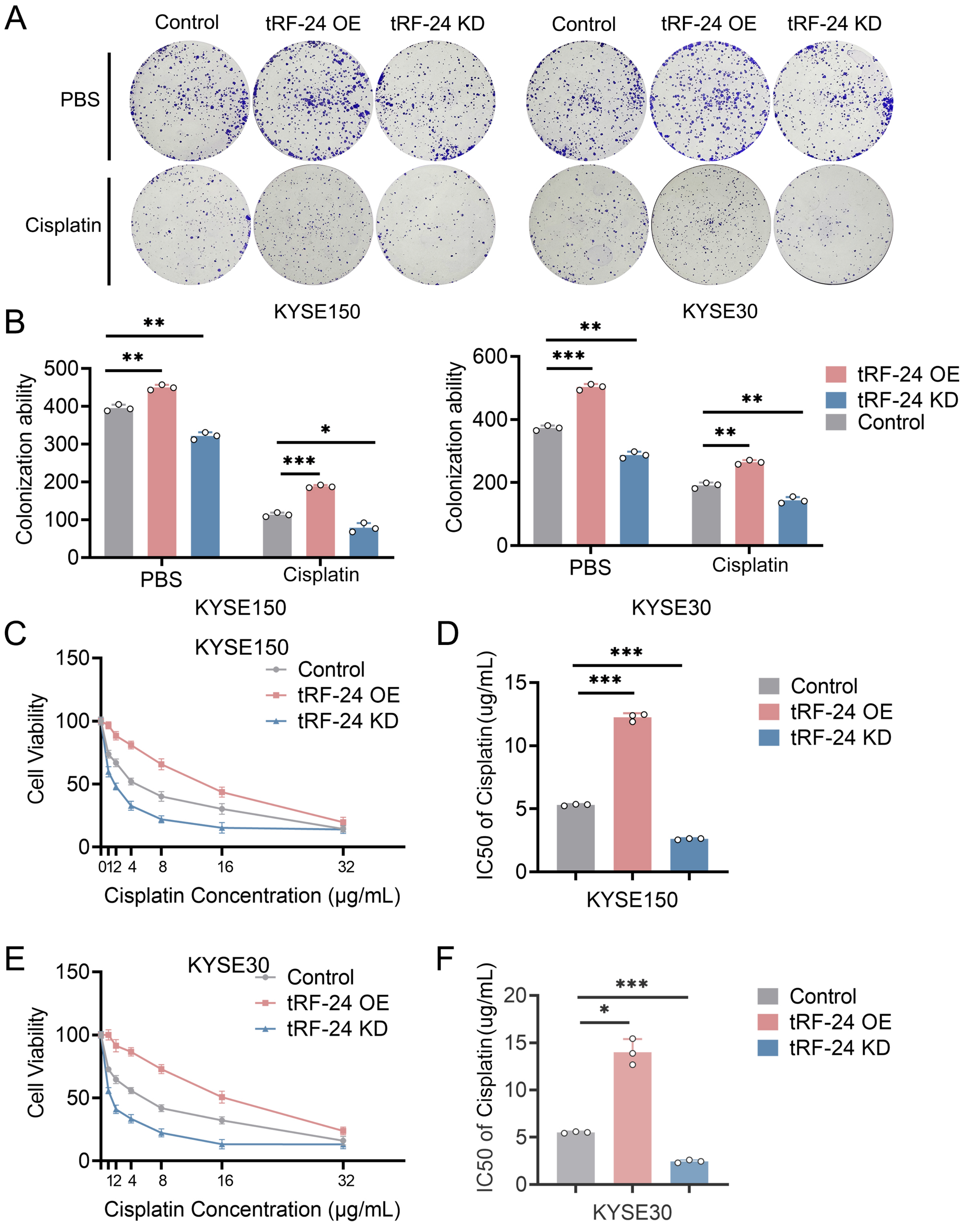
A
Control
tRF-24 OE
tRF-24 KD
Control
tRF-24 OE
tRF-24 KD
PBS
Cisplatin
KYSE150
KYSE30
B
Colonization ability
PBS
Cisplatin
tRF-24 OE
tRF-24 KD
Control
KYSE150
KYSE30
C
KYSE150
Cell Viability
Control
tRF-24 OE
tRF-24 KD
Cisplatin Concentration (μg/mL)
D
IC50 of Cisplatin(ug/mL)
Control
tRF-24 OE
tRF-24 KD
KYSE150
E
KYSE30
Cell Viability
Control
tRF-24 OE
tRF-24 KD
Cisplatin Concentration (μg/mL)
F
IC50 of Cisplatin(ug/mL)
Control
tRF-24 OE
tRF-24 KD
KYSE30

Figure S9

Supplementary Figure 9. Effects of *tRF-24* on cisplatin chemoresistance in ESCC cells.

(**A-B**) Representative images (**A**) and colony numbers (**B**) in KYSE150 and KYSE30 cells treated with 1 μg/mL cisplatin and PBS. Colonies containing more than 50 cells were counted. (**C-F**) Cell viability (**C, E**) and IC_{50} of cisplatin (**D, F**) were assessed after 48 h in KYSE150 and KYSE30 cells with control, *tRF-24* overexpression (OE) or knockdown (KD) conditions. Data are presented as mean ± SEM from 3 independent experiments. $^{*}P < 0.05$, $^{**}P < 0.01$, $^{***}P < 0.001$ by one-way ANOVA with Dunnett's T3 multiple comparison test.

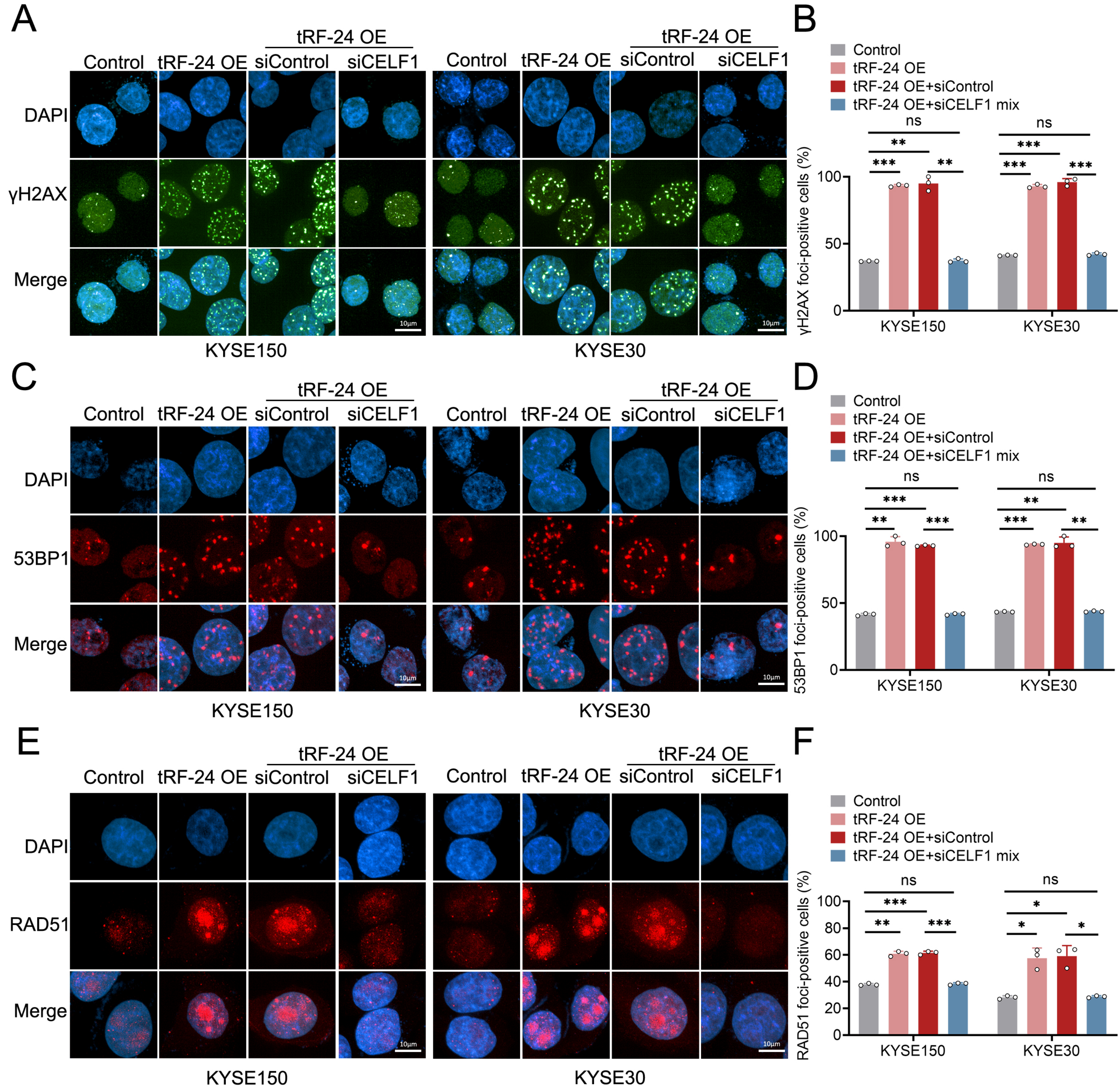
A
tRF-24 OE
Control
tRF-24 OE
siControl
siCELF1
DAPI
γH2AX
Merge
10μm
KYSE150
KYSE30
B
Control
tRF-24 OE
tRF-24 OE+siControl
tRF-24 OE+siCELF1 mix
ns
**
***
γH2AX foci-positive cells (%)
100
50
0
KYSE150
KYSE30
C
53BP1
D
53BP1 foci-positive cells (%)
E
RAD51
F
RAD51 foci-positive cells (%)
80
60
40
20
*

Figure S10

Supplementary Figure 10. CELF1 knockdown rescues *tRF-24* induced DSB repair in ESCC cells.

(**A-F**) Representative IF images showing the expression of γH2AX (**A-B**), 53BP1 (**C-D**) and RAD51 (**E-F**) following CELF1 silencing in ESCC cells with *tRF-24* overexpression after 24 h of treatment with 4 μM cisplatin. The percentages of cells with positive nuclei (defined as ≥10 foci per nucleus for γH2AX and 53BP1; ≥5 foci per nucleus for RAD51) are quantified for each cell line. Scale bars: 10 μm. Data are presented as mean ± SEM from 3 independent experiments. ns, no significance, $*P < 0.05$, $**P < 0.01$, $***P < 0.001$ by one-way ANOVA with Dunnett's T3 multiple comparison test.

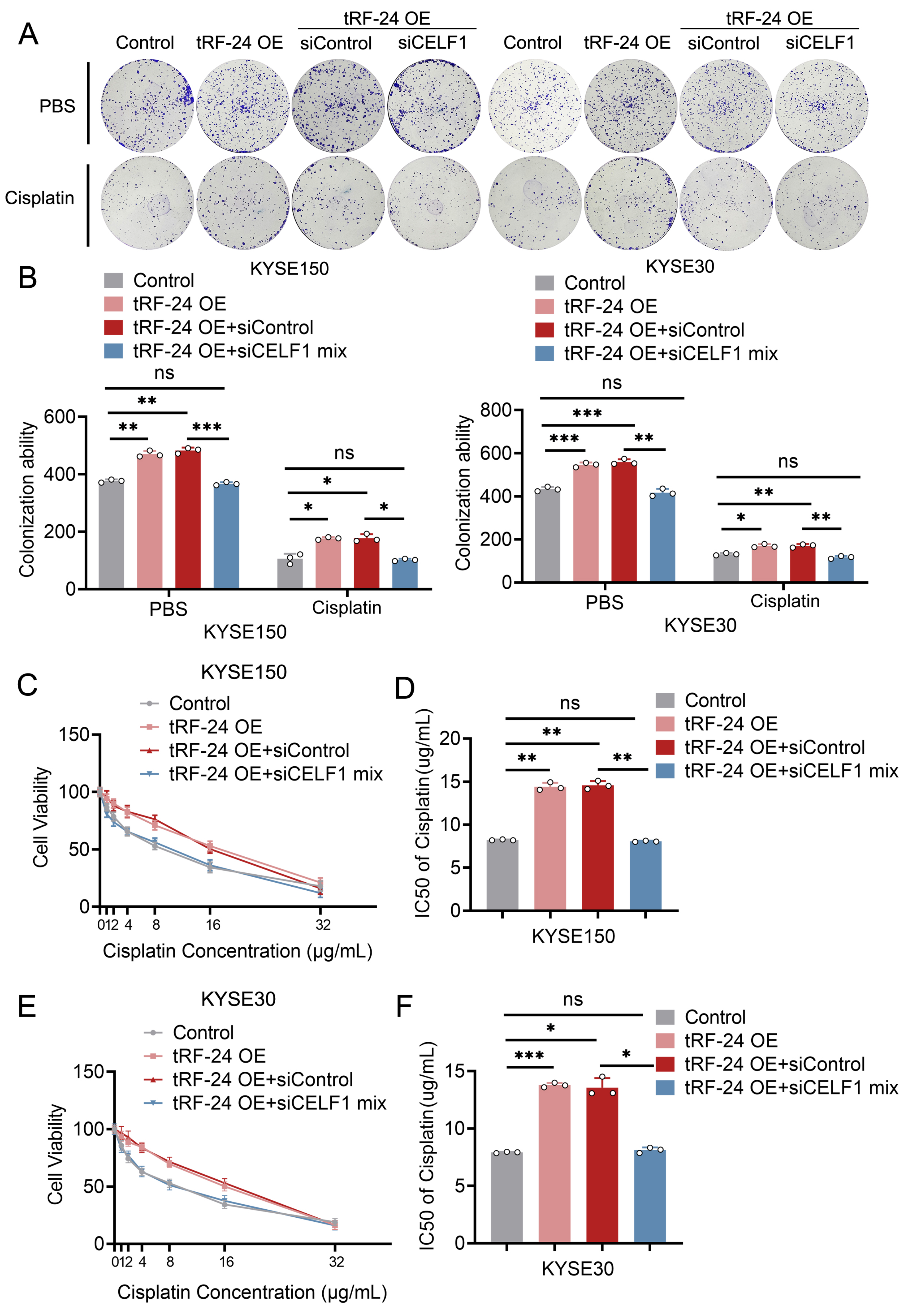

A
Control
tRF-24 OE
tRF-24 OE
siControl
siCELF1
PBS
Cisplatin
KYSE150
KYSE30
B
Control
tRF-24 OE
tRF-24 OE+siControl
tRF-24 OE+siCELF1 mix
ns
Colonization ability
PBS
Cisplatin
C
Cell Viability
Cisplatin Concentration (μg/mL)
D
IC50 of Cisplatin (ug/mL)
E
F

Figure S11

Supplementary Figure 11. CELF1 knockdown rescues *tRF-24* induced cisplatin chemoresistance in ESCC cells.

(**A-B**) The effect of CELF1 knockdown on colony formation ability in KYSE150 and KYSE30 cells under *tRF-24*-overexpressing conditions and treated with 1 μg/mL cisplatin. Representative images (**A**) and colony numbers (**B**) are shown. Colonies containing >50 cells were counted. (**C-F**) Cell viability (**C, E**) and IC_{50} of cisplatin (**D, F**) were assessed after 48 h in KYSE150 and KYSE30 cells with control, *tRF-24* overexpression (OE) or knockdown (KD) conditions. Data are presented as mean ± SEM from 3 independent experiments. ns, no significance, *$P < 0.05$, **$P < 0.01$, ***$P < 0.001$ by one-way ANOVA with Dunnett's T3 multiple comparison test.

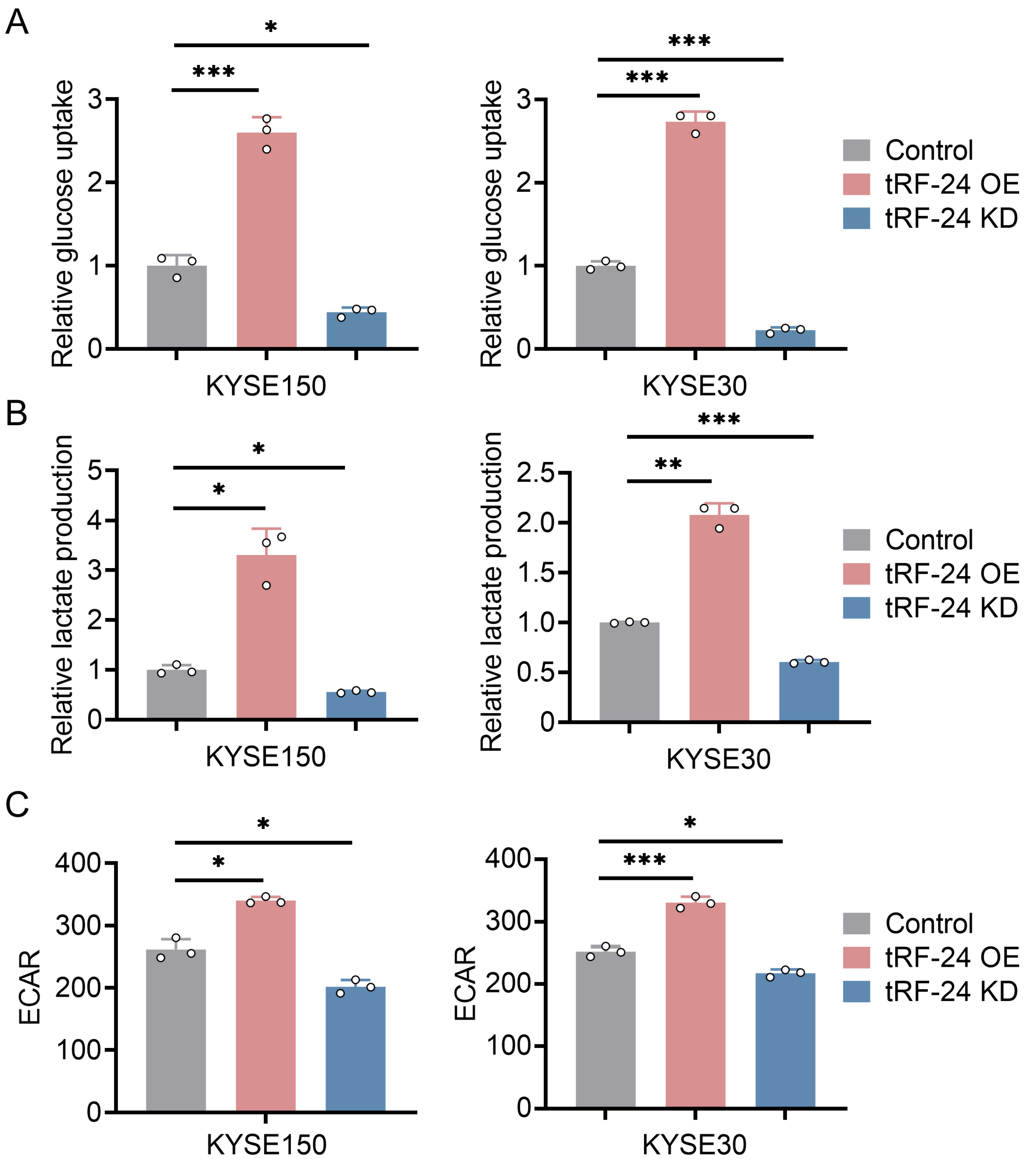
A
Relative glucose uptake
KYSE150
KYSE30
Control
tRF-24 OE
tRF-24 KD
B
Relative lactate production
C
ECAR
Figure S12

Supplementary Figure 12. Effects of *tRF-24* on glycolysis in ESCC cells.

(**A-C**) After overexpressing or knocking down *tRF-24*, the glucose uptake rates (**A**), lactic acid production (**B**) and extracellular acidification rate (ECAR) (**C**) in KYSE150 and KYSE30 cells were detected. Data are presented as mean ± SEM from 3 independent experiments. $^{*}P < 0.05$, $^{**}P < 0.01$, $^{***}P < 0.001$ by one-way ANOVA with Dunnett's T3 multiple comparison test.

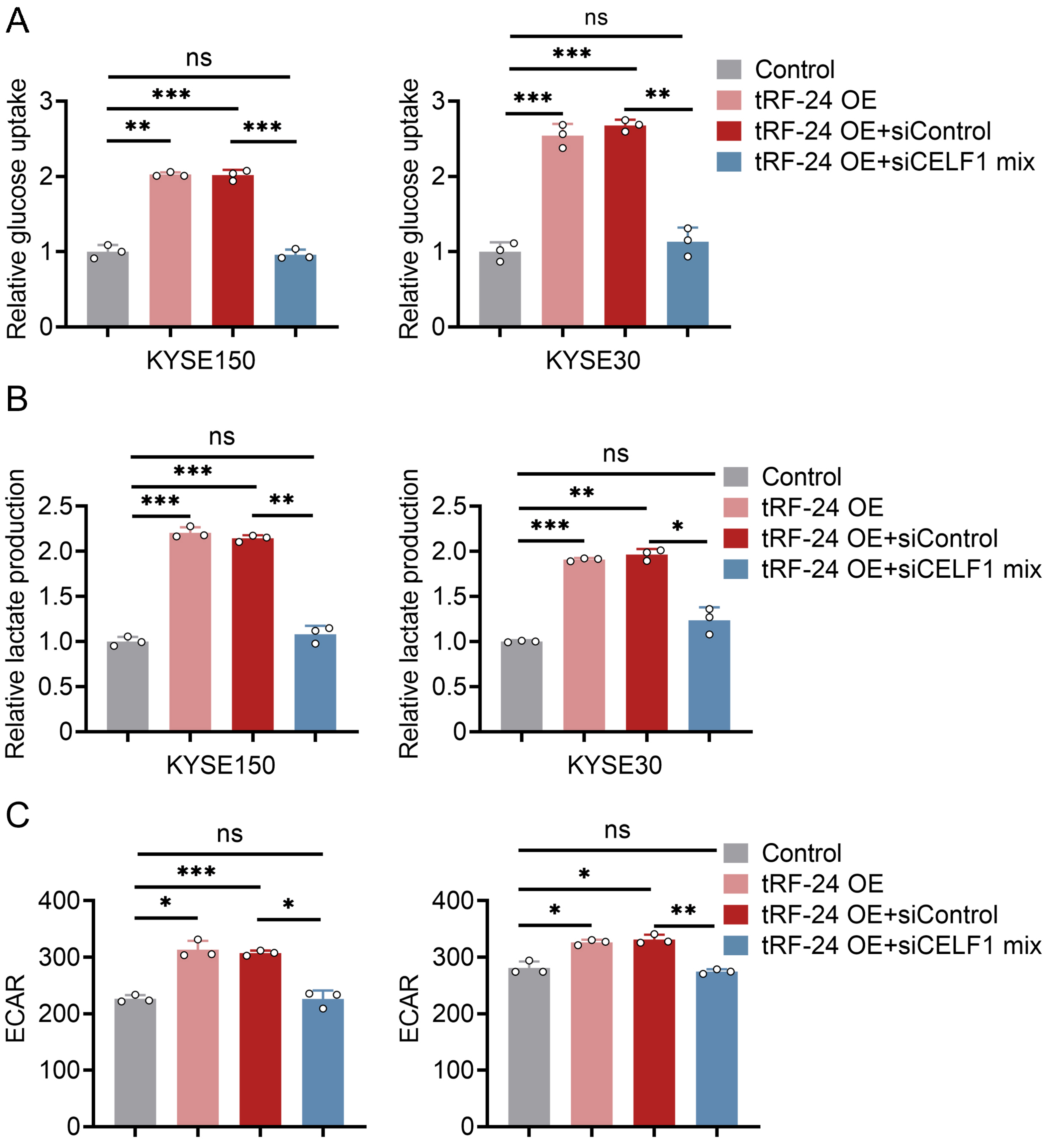

A
ns
***
**
***
3
2
1
0
Relative glucose uptake
KYSE150
ns
***
***
**
Relative glucose uptake
KYSE30
Control
tRF-24 OE
tRF-24 OE+siControl
tRF-24 OE+siCELF1 mix
B
ns
***
***
**
2.5
2.0
1.5
1.0
0.5
0
Relative lactate production
KYSE150
ns
**
***
*
Relative lactate production
KYSE30
Control
tRF-24 OE
tRF-24 OE+siControl
tRF-24 OE+siCELF1 mix
C
ns
***
*
*
400
300
200
100
0
ECAR
KYSE150
ns
*
*
**
ECAR
KYSE30
Control
tRF-24 OE
tRF-24 OE+siControl
tRF-24 OE+siCELF1 mix

Figure S13

Supplementary Figure 13. CELF1 knockdown rescues *tRF-24* induced glycolysis in ESCC cells.

(**A-C**) Effects of CELF1 knockdown on the glucose uptake rates (**A**), lactic acid production (**B**) and extracellular acidification rate (ECAR) (**C**) in cells with *tRF-24* overexpression. Data are presented as mean ± SEM from 3 independent experiments. ns, no significance, $^{*}P < 0.05$, $^{**}P < 0.01$, $^{***}P < 0.001$ by one-way ANOVA with Dunnett's T3 multiple comparison test.

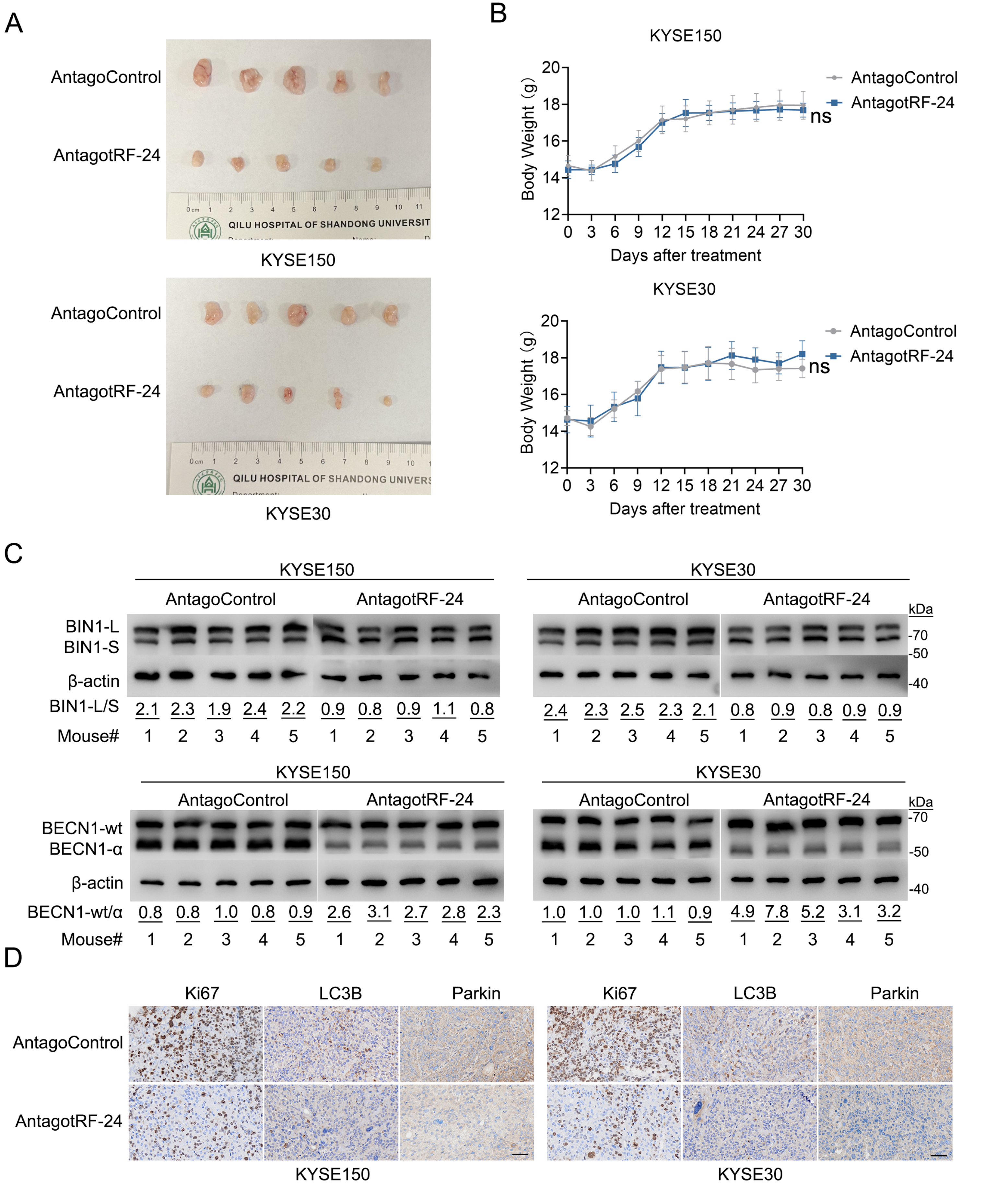
A
AntagoControl
AntagotRF-24
KYSE150
AntagoControl
AntagotRF-24
KYSE30
B
KYSE150
Body Weight (g)
Days after treatment
AntagoControl
AntagotRF-24
ns
KYSE30
C
KYSE150
KYSE30
AntagoControl
AntagotRF-24
BIN1-L
BIN1-S
β-actin
BIN1-L/S
Mouse#
kDa
BECN1-wt
BECN1-α
BECN1-wt/α
D
Ki67
LC3B
Parkin

Figure S14

Supplementary Figure 14. Therapeutic effects of antagotRF-24 on subcutaneous xenografts in mice.

(**A**) Images of mice with subcutaneous xenografts treated with antagotRF-24 or antagoControl (5 mice per group). (**B**) Body weight gain of mice carrying subcutaneous xenografts treated with antagotRF-24 or antagoControl. Data shown as mean ± SEM, n = 5. ns, not significant difference by Student's t test. (**C**) Western blot analysis of BIN1-L, BIN1-S, BECN1-wt and BECN1-α expression in subcutaneous xenografts of mice. (**D**) Immunohistochemical staining of Ki67, LC3B and Parkin in subcutaneous xenografts from mice treated with antagotRF-24 or antagoControl. Scale bars: 50 μm.

Supplementary Table 1. Baseline demographics and clinical characteristics of individuals with ESCC.

	Stable (n = 74)	Relapsed (n = 22)
Age, mean (SEM[a])	64.4 (0.9)	64.5 (1.9)
Sex, *n* (%)		
Male	59 (79.7)	17 (77.3)
Female	15 (20.3)	5 (22.7)
Differentiation, *n* (%)		
Well	7 (9.5)	0 (0.0)
Moderate	49 (66.2)	16 (72.7)
Poor	15 (20.3)	5 (22.7)
Unknown	3 (4.1)	1 (4.5)
Lymph node metastasis, *n* (%)		
Positive	32 (43.2)	19 (86.4)
Negative	42 (56.8)	3 (13.6)
Intravascular tumor thrombus, *n* (%)		
Yes	16 (21.6)	8 (36.4)
No	51 (68.9)	11 (50.0)
Unknown	7 (9.5)	3 (13.6)
Neural invasion, *n* (%)		
Yes	11 (14.9)	7 (31.8)
No	56 (75.7)	12 (54.5)
Unknown	7 (9.5)	3 (13.6)
TNM stage[b], *n* (%)		
I	13 (17.6)	0 (0.0)
II	29 (39.2)	4 (18.2)
III	31 (41.9)	17 (77.3)
IV	1 (1.4)	1 (4.5)
Smoking status, *n* (%)		
Ever	46 (62.2)	15 (68.2)
Never	24 (32.4)	7 (31.8)
Unknown	4 (5.4)	0 (0.0)
Drinking status, *n* (%)		
Ever	48 (64.9)	16 (72.7)
Never	21 (28.4)	5 (22.7)
Unknown	5 (6.8)	1 (4.5)
Treatment, n (%)		
Surgery only	54 (73.0)	11 (50.0)
Surgery + neo-adjuvant therapy	2 (2.7)	0 (0.0)
Surgery + radiotherapy / chemotherapy / Targeted therapy / Immunotherapy	18 (24.3)	11 (50.0)

[a]SEM, standard error of mean.

[b]Tumor TNM staging were reviewed by at least 3 pathologists and defined according to the American Joint Committee on Cancer (AJCC) 8th edition.

Supplementary Table 2. Upregulated tsRNAs in ESCC tumors compared to normal tissues (Fold Change > 2 and *P* value < 0.05, GSE207635 data).

NO.	tsRNA symbol	log_2FC	*P* value
1	tRF-32-FP18LPMBQ4NKJ	5.284114682	2.49E-05
2	tRF-24-RPM8309M2S	5.063835873	0.006649
3	tRF-30-Q99P9P9NH57S	4.572274989	0.000339
4	tRF-34-PSQP4PW3FJI0EJ	4.231960335	4.50E-07
5	tRF-34-R9JP9P9NH5SYHQ	4.066335849	2.26E-08
6	tRF-32-R29P4P9L5HJVQ	4.051599879	0.001133
7	tRF-31-FP18LPMBQ4NKD	3.992503756	3.07E-05
8	tRF-34-Q99P9P9NH57S15	3.672620029	3.15E-07
9	tRF-33-P4R8YP9LON4VDP	3.357910667	9.29E-06
10	tRF-34-PW5SVP9N15WV2P	3.295250876	3.99E-05
11	tRF-16-RP9830D	3.260061429	0.004002
12	tRF-34-PS5P4PW3FJHPEQ	3.250895401	1.42E-05
13	tRF-29-79MP9PMNH5F5	3.232515774	0.000342
14	tRF-28-86J8WPMN1E0J	3.181347609	0.000134
15	tRF-29-87R8WP9I1EJ4	3.145493144	0.000119
16	tRF-34-79MP9P9NH57S15	3.133305148	2.54E-05
17	tRF-29-87R8WP9N1EJ4	3.116158047	3.64E-07
18	tRF-29-Q99P9P9NH525	3.097597127	7.72E-05
19	tRF-34-79MP9P9NH57S1O	3.025847799	0.000939
20	tRF-34-PSQP4PW3FJIKE5	3.019294251	3.05E-06
21	tRF-32-87R8WP9I1EWJM	2.943439558	1.84E-05
22	tRF-28-Q99P9P9NH50E	2.89748544	0.012094
23	tRF-16-RPM830E	2.862142632	1.16E-05
24	tRF-30-87R8WP9N1EWJ	2.76412572	3.51E-05
25	tRF-30-QNR8VP9NFQFY	2.700945433	0.000878
26	tRF-34-897PVP941QKS1P	2.683528624	0.00098

27	tRF-31-PSQP4PW3FJI0B	2.664288141	0.000136
28	tRF-33-FP18LPMBQ4NKDJ	2.625127014	0.047414
29	tRF-32-PSQP4PW3FJIK1	2.596011165	0.000487
30	tRF-28-PW5SVP9N1503	2.568686319	0.002398
31	tRF-16-RPM830D	2.555806595	4.87E-05
32	tRF-34-86J8WPMN1E8Y2Q	2.55259093	0.00097
33	tRF-29-PW5SVP9N15JP	2.535462514	0.000132
34	tRF-28-87R8WP9N1E0K	2.526964051	2.75E-05
35	tRF-32-87R8WP9N1EWJM	2.489566068	2.57E-05
36	tRF-34-86V8WPMN1E8Y2Q	2.44546789	0.002676
37	tRF-31-86J8WPMN1E8Y0	2.401858494	4.45E-05
38	tRF-32-P4R8YP9LON4V3	2.33546093	0.000337
39	tRF-31-86V8WPMN1E8Y0	2.321809764	0.000697
40	tRF-30-PSQP4PW3FJI0	2.321724582	0.00031
41	tRF-28-86V8WPMN1E0J	2.321038019	0.005682
42	tRF-30-86J8WPMN1E8Y	2.301035435	0.000111
43	tRF-29-P4R8YP9LONHK	2.300353545	8.09E-05
44	tRF-30-PW5SVP9N15WV	2.26804549	1.42E-05
45	tRF-16-S3M830E	2.262643302	0.002578
46	tRF-30-QNR8VP94FQFY	2.259699671	0.006947
47	tRF-32-PW5SVP9N15WVN	2.251532514	0.000554
48	tRF-34-PSQP4PW3FJI0E5	2.249272674	0.000407
49	tRF-16-OUR830E	2.208275965	0.023016
50	tRF-16-SP5830D	2.178334089	0.010722
51	tRF-30-87R8WP9I1EWJ	2.160589965	0.011773
52	tRF-28-PSQP4PW3FJD0	2.15745478	0.003461
53	tRF-32-PSQP4PW3FJI01	2.119289704	0.001008
54	tRF-32-ROD8N0X0JYOYO	2.105684501	0.017429
55	tRF-31-87R8WP9N1EWJ0	2.036853303	0.000711

56	tRF-33-Q1Q89P9L842205	2.005749359	0.004065
57	tRF-33-PNR8YP9LON4VDP	1.875238178	0.008567
58	tRF-29-PSQP4PW3FJFL	1.848596153	0.006902
59	tRF-34-87R8WP9I1EWJIQ	1.84509942	0.007318
60	tRF-33-PNR8YP9LON4VD5	1.844451438	0.003745
61	tRF-32-Q99P9P9NH57SJ	1.815722917	0.000315
62	tRF-32-PS5P4PW3FJHP1	1.802898261	0.00501
63	tRF-28-P4R8YP9LOND5	1.710906599	0.010672
64	tRF-29-86J8WPMN1EJ3	1.689413702	0.020408
65	tRF-30-86V8WPMN1E8Y	1.581475291	0.046313
66	tRF-31-Q99P9P9NH57SD	1.545595226	0.022144
67	tRF-34-87R8WP9N1EWJI5	1.534518612	0.037498
68	tRF-32-6978WPRLXN4VQ	1.519871082	0.011673
69	tRF-29-PSQP4PW3FJF4	1.489145055	0.040576
70	tRF-34-79MP9PMNH5IS15	1.451963321	0.024132
71	tRF-32-PNR8YO9LON4V3	1.317879517	0.03607
72	tRF-29-PNR8YP9LONHK	1.302715186	0.029587
73	tRF-31-P4R8YP9LON4VD	1.302276311	0.036941
74	tRF-31-PS5P4PW3FJHPB	1.298378817	0.047572
75	tRF-30-P4R8YP9LON4V	1.290436502	0.037715
76	tRF-31-PW5SVP9N15WV0	1.152450626	0.046189

Supplementary Table 3. Primers and probes used in this study.

**RT-qPCR Primer**		
**Gene Symbol**	**Sequence (5’ → 3’)**	
	Forward	Reverse
CELF1	AGAGTATTGGTGCTGCTGGAAG	AACCTTGGCAGACACGACATT
BECN1-wt	GTGGCTTTCCTGGACTGTGTG	CAGCATGAACTTGAGAGCTTTTG
BECN1-α	TCTGACAGACAAATCTAAGGATGGA	TCGTCAGCATGAACTTGAGAGC
BIN1-L	GAACAAGAGCCCTTCGCCT	TCTGCTCCTGCTTGACTTCCT
BIN1-S	GAGATCAGAGTCAACCACGAGC	GGCCTCTGCTGGCTGAGAT
GAPDH	TTGGCCAGGGGTGCTAAG	AGCCAAAAGGGTCATCATCTC
ACTB	CAGGGCGTGATGGTGGGCATG	GTAGAAGGTGTGGTGCCAGATT
**Northern blot probe (DIG-labeled)**	**Sequence (5’ → 3’)**	
tRF-24-RPM8309M2S	CUUAGACCACUCGGCCACGCUACC	
**RNA pull-down probe (3’-biotin)**	**Sequence (5’ → 3’)**	
non-targeting oligo	UUGUACUACACAAAAGUACUG	
tRF-24-RPM8309M2S sense	GGUAGCGUGGCCGAGUGGUCUAAG	
tRF-24-RPM8309M2S antisense	CUUAGACCACUCGGCCACGCUACC	
**RNA FISH probe (3’-biotin)**	**Sequence (5’ → 3’)**	
tRF-24-RPM8309M2S antisense	CUUAGACCACUCGGCCACGCUACC	

**RNA sequence for *in vitro* kinase assay**	**Sequence (5’ → 3’)**
tRF-24-RPM8309M2S sense	GGUAGCGUGGCCGAGUGGUCUAAG
tRF-24-RPM8309M2S antisense	CUUAGACCACUCGGCCACGCUACC

**RT-qPCR primer for tRFs**	**Sequence (5’ → 3’)** Forward	Reverse
tRF-24-RPM8309M2S	TATCTGGTAGCGTGGCCGAGT	TATCCTTCTTCACGACTCCTTCAC
tRF-32-FP18LPMBQ4NKJ	CGCAGCGGAAGCGTGC	AGTGCAGGGTCCGAGGTATT
tRF-30-Q99P9P9NH57S	CGGCTTCTGTAGTGTAGTGGTTATCA	AGTGCAGGGTCCGAGGTATT
tRF-34-PSQP4PW3FJI0EJ	GCTAGCTCAGTCGGTAGAGCATG	AGTGCAGGGTCCGAGGTATT
tRF-34-R9JP9P9NH5SYHQ	TCCATAGTGTAGTGGTTATCACGTC	AGTGCAGGGTCCGAGGTATT
tRF-32-R29P4P9L5HJVQ	GGGGATGTAGCTCAGTGGTAGA	AGTGCAGGGTCCGAGGTATT
tRF-31-FP18LPMBQ4NKD	GCGCAGCGGAAGCGTG	AGTGCAGGGTCCGAGGTATT
tRF-34-Q99P9P9NH57S15	CTTCTGTAGTGTAGTGGTTATCACGTT	AGTGCAGGGTCCGAGGTATT
tRF-33-P4R8YP9LON4VDP	CATGGGTGGTTCAGTGGTAGAA	AGTGCAGGGTCCGAGGTATT
tRF-34-PW5SVP9N15WV2P	GCCGTGATCGTATAGTGGTTAGTAC	AGTGCAGGGTCCGAGGTATT
tRF-16-RP9830D	CGCGCGGGTAGTGTGG	AGTGCAGGGTCCGAGGTATT
tRF-34-PS5P4PW3FJHPEQ	CCCGGATAGCTCAGTCGGTA	AGTGCAGGGTCCGAGGTATT
tRF-29-79MP9PMNH5F5	GTTTCCGTAGTGTAGCGGTTATC	AGTGCAGGGTCCGAGGTATT
tRF-28-86J8WPMN1E0J	CCCACATGGTCTAGCGGTTAG	AGTGCAGGGTCCGAGGTATT
tRF-29-87R8WP9I1EJ4	CCTGGTGGTCTAGTGGCTAGG	AGTGCAGGGTCCGAGGTATT

U6	CTCGCTTCGGCAGCACA	AACGCTTCACGAATTTGCGT

Supplementary Table 4. Plasmids used in this study.

Descriptions	Source	Product number
pCMV-mCherry-GFP-LC3B	Beyotime	D2816
pcDNA3.1-EGFP-CELF1 (FL: 1-486)	This paper	N/A
pcDNA3.1-EGFP-CELF1 (RRM1:13-102)	This paper	N/A
pcDNA3.1-EGFP-CELF1 (RRM2: 105-191)	This paper	N/A
pcDNA3.1-EGFP-CELF1 (DI/RRM3: 273-486)	This paper	N/A
pcDNA3.1-EGFP-CELF1 (RRM3: 398-486)	This paper	N/A
pcDNA3.1-3×flag-CELF1 (WT)	This paper	N/A
pcDNA3.1-3×flag-CELF1 (T26A)	This paper	N/A
pcDNA3.1-3×flag-CELF1 (S28A)	This paper	N/A
pcDNA3.1-3×flag-CELF1 (S52A)	This paper	N/A
Penter-3×flag-6×his-AKT1	This paper	CH846646
Pet28a-6×his-EGFP-CELF1-6×his	This paper	N/A
Pet28a-6×his-EGFP-CELF1 (ΔIDR)-6×his	This paper	N/A
pcDNA3.1-EGFP-CELF1 (ΔIDR)	This paper	N/A

Supplementary Table 5. Sequences of siRNAs or mimics used in this study.

siRNAs	Sequence (5’ → 3’)
siControl	UUCUCCGAACGUGUCACGUTT
siCELF1#1	UAAUGCAGCUUUACGGGUGTT
siCELF1#2	AUUACUGCUGCUAGAGCUAGGTT
siAKT1#1	AGGAAGUCAUCGUGGCCAATT
siAKT1#2	GCACUUUCGGCAAGGUGAUTT
**Mimics**	**Sequence (5’→ 3’)**
tRF-24-RPM8309M2S Control	UCUACUCUUUCUAGGAGGUUGUGA
tRF-24-RPM8309M2S mimics	GGUAGCGUGGCCGAGUGGUCUAAG
tRF-24-RPM8309M2S inhibitor	CUUAGACCACUCGGCCACGCUACC
AntagoControl	UCUACUCUUUCUAGGAGGUUGUGA
AntagotRF-24-RPM8309M2S	CUUAGACCACUCGGCCACGCUACC

Supplementary Table 6. Antibodies used in this study.

Protein name	Host species	Product number
CELF1	M	Santa Cruz Biotechnology, sc-20003
p-Ser28-CELF1	R	Antagene, Phospho- AB2A181
MYO1C	M	Santa Cruz Biotechnology, sc-136544
SARS2	R	Abcam, ab154825
BSG	R	Abcam, ab108308
SET	R	Abcam, ab181990
AKT1	M	Santa Cruz Biotechnology, sc-5298
LC3B	R	HUABIO, ET1701-65
P62	R	HUABIO, HA721171
Parkin	R	HUABIO, ET1702-60
PINK1	R	HUABIO, ER1706-27
TOMM20	R	HUABIO, ET1609-25
BECN1	M	Santa Cruz Biotechnology, sc-48381
BIN1	M	Santa Cruz Biotechnology, sc-13575
Ki67	R	HUABIO, HA721115
GFP	R	HUABIO, SY0243
FLAG	M	Sigma, F1804
HA	M	Sigma, SAB2702196
β-ACTIN	M	HUABIO, M1210-2
GAPDH	M	Proteintech, 60004-1-Ig
Histone H3	R	HUABIO, M1309-1

M, Mouse; R, Rabbit

Table S7. Antibodies used in this study

M, Mouse; R, Rabbit

Protein name	Host species	Product number
CELF1	M	Santa Cruz Biotechnology, sc-20003
MYO1C	M	Santa Cruz Biotechnology, sc-136544
SARS2	R	Abcam, ab154825
BSG	R	Abcam, ab108308
SET	R	Abcam, ab181990
GAPDH	M	Proteintech, 60004-1-Ig
GFP	R	HUABIO, SY0243
FLAG	M	Sigma, F1804
HA	M	Sigma, SAB2702196
AKT1	M	Santa Cruz Biotechnology, sc-5298
LC3B	R	HUABIO, ET1701-65
P62	R	HUABIO, HA721171
Parkin	R	HUABIO, ET1702-60
PINK1	R	HUABIO, ER1706-27
TOMM20	R	HUABIO, ET1609-25
β-ACTIN	M	HUABIO, M1210-2
P-Ser28-CELF1	R	Antagene, Phospho- AB2A181
Histone H3	R	HUABIO, M1309-1
BECN1	M	Santa Cruz Biotechnology, sc-48381
BIN1	M	Santa Cruz Biotechnology, sc-13575
Ki67	R	HUABIO, HA721115
Vimentin	R	Abclonal, A19607
N-Cadherin	R	Abclonal, A19083
E-Cadherin	R	HUABIO, ET1607-75
Snail	R	HUABIO, ER1706-22
p-Histone H2A.X (S139)	M	Abways, AB3322
RAD51	R	HUABIO, ET1705-96
53BP1	R	HUABIO, ET1704-05

Supplementary Table 8. RNA-seq identification of genes involved in the alternative splicing of CELF1.

No.	Gene Names	No.	Gene Names	No.	Gene Names
1	ABCA11P	85	GIGYF2	169	RABGAP1L
2	ABI1	86	GMPPA	170	RAD52
3	ANAPC11	87	GNPDA2	171	REPIN1
4	ANK3	88	GOLGB1	172	REXO5
5	ANKRD33B	89	GSE1	173	RO60
6	ANKRD36	90	GXYLT2	174	ROBO3
7	ANKS3	91	HERC2P9	175	RPAIN
8	ANXA2	92	HLA-F-AS1	176	RWDD3
9	ARID5A	93	HM13	177	SCAPER
10	ARIH2	94	HMBOX1	178	SEMA4D
11	ARL13B	95	HNRNPA2B1	179	SERPINH1
12	ARMC9	96	HNRNPH1	180	SETD5
13	ASTE1	97	HPS4	181	SF3B1
14	ASXL1	98	HSD3B7	182	SFI1
15	ATF6	99	HSF1	183	SGO1
16	ATRX	100	HUWE1	184	SH2B1
17	ATXN2	101	IGSF1	185	SLC12A2-DT
18	BECN1	102	IL24	186	SLC12A4
19	BIN1	103	IP6K2	187	SLC22A5
20	BTN3A2	104	IQSEC1	188	SLC25A26
21	C18orf25	105	ITGB1BP1	189	SLC25A35
22	C1orf116	106	KDM6A	190	SLC38A9
23	C2CD5	107	KIF1B	191	SLC44A3-AS1
24	CALM1	108	KLC1	192	SLMAP
25	CASP8	109	LAMA5	193	SMC4
26	CBWD3	110	LINC00630	194	SNHG26

27	CCDC18	111	LINC00958	195	SNHG32
28	CDK11B	112	LPIN1	196	SNX14
29	CENPT	113	LRPPRC	197	SPIDR
30	CEP126	114	LRRC28	198	SSBP3
31	CEP192	115	LRRC40	199	SSR2
32	CEP290	116	LTBP4	200	STARD3NL
33	CEP57L1	117	MAP7D1	201	SUMF2
34	CHD9	118	MBOAT2	202	SUN1
35	COL7A1	119	MCEE	203	SUPT5H
36	CRYBG2	120	MCM8	204	SUZ12P1
37	CSGALNACT2	121	MDC1	205	TAF1D
38	DAGLA	122	MDN1	206	TENM2
39	DAXX	123	MIR4435-2HG	207	TET2
40	DCAF8	124	MPPE1	208	TGIF2
41	DDR1	125	MROH6	209	TMEM116
42	DEPDC5	126	MTO1	210	TMEM143
43	DGUOK	127	MTUS1	211	TMEM14B
44	DLG1	128	MUC16	212	TMEM150A
45	DMD	129	MUC20-OT1	213	TMEM192
46	DMPK	130	MYO9B	214	TPT1-AS1
47	DNM1	131	N4BP2L2	215	TRA2B
48	DOP1A	132	NAA25	216	TRAPPC3
49	DPH5-DT	133	NARS2	217	TRMT2B
50	DUXAP9	134	NCOR2	218	TSEN2
51	DYRK3	135	NDUFAF6	219	TSG101
52	EDIL3-DT	136	NEDD4L	220	TTC3
53	ELMO2	137	NENF	221	TUBGCP6
54	ELMOD3	138	NFATC2IP	222	TYK2

55	ENSG00000233928	139	NMRAL1	223	UBE2Q2
56	ENSG00000244560	140	NSD2	224	UBE3A
57	ENSG00000259316	141	NSUN5P1	225	UBIAD1
58	ENSG00000268750	142	NSUN6	226	UBR3
59	ENSG00000273066	143	NT5C	227	UHRF2
60	ENSG00000275496	144	NTAN1	228	USP53
61	ENSG00000284879	145	NUBP2	229	VEZT
62	ENSG00000285756	146	NUP62	230	VPS13D
63	ENSG00000286104	147	PAK4	231	VPS8
64	ENSG00000287908	148	PATZ1	232	WASH7P
65	ENSG00000288473	149	PDCD2L	233	WBP1
66	ENSG00000288632	150	PDIK1L	234	WDR27
67	ENSG00000289758	151	PDP1	235	WDSUB1
68	EPB41L2	152	PELI3	236	YDJC
69	ETFDH	153	PHACTR4	237	ZBTB34
70	FAM111A	154	PIEZO1	238	ZBTB8OS
71	FAM13B	155	PIF1	239	ZDHHC16
72	FAM86DP	156	PIK3CB	240	ZFAND2A-DT
73	FANCA	157	PLCB4	241	ZMYM5
74	FER1L4	158	PLEKHA7	242	ZNF195
75	FGGY	159	POLG	243	ZNF221
76	FNBP1	160	POLL	244	ZNF236
77	FNIP1	161	PPM1K	245	ZNF433-AS1
78	FRG1HP	162	PPP6R2	246	ZNF678
79	GALNT11	163	PROM2	247	ZNF691
80	GAPDH	164	PRRC2B	248	ZNF721
81	GARS1-DT	165	PTGES3L-AARSD1	249	ZNF79
82	GAS5	166	PTK2	250	ZSWIM8

83	GEMIN7	167	PUM2	
84	GGA1	168	RAB4A	
